# Part II. Spirit of the Foreign Periodicals

**Published:** 1834-04-01

**Authors:** 


### Spirit of the Foreign Periodicals, &c.

I. De Fistulis Colli Congenitis, adjecta Fissurarum Branchialium in Mammalibus, avibusque Historia succincta. — Berolini, 1832.

M. Ascherson, the author of this memoir, has called the attention of his medical brethren to a curious congenital anomaly, or "vitium formationis," which he has observed in several persons. The number of cases altogether amounts to eleven; and most of them occurred in female children, of a scrofulous, or at least of a lymphatic constitution. The authenticity of most is guaranteed by the testimony of that able physiologist Rudolphi. The following may be given as a brief description of the disease:—On the anterior and lateral part of the neck there is observed a fistulous opening, which is situated generally in that triangular hollow between the clavicle and the two points of insertion of the sterno-mastoideus; but sometimes it is at the inner edge of this muscle. It is much more frequently found on the right than on the left side; and if there should happen to be one on either side, that on the right is always larger and placed somewhat higher up than the other one. The aperture is invariably very narrow; occasionally scarcely visible, but at other times it is surrounded with a red circle, or it may project like a papilla. It generally follows the movements of the pharynx in deglutition, and when this is the case, we observe a transverse furrow, at the bottom of which is situated the fistulous opening. If a probe be introduced, it may perhaps be pushed forwards a little way, but in most of the cases it is stopped very soon, in consequence of the sinuosity of the canal. In one case, fluid injected at the outer opening, passed into the pharynx, and the patient was sensible of its taste; and in another, the attempts made to cure the fistula in this way were followed by disagreeable consequences, such as swelling of the neck, smarting pain, and the sensation as if a foreign body was sticking in the pharynx. On no occasion was any air ever observed to escape from the opening, even when the effort of expiration was strong, while the mouth and nostrils were kept closed. The discharge from the fistula was sometimes viscid and clear, and at other times, more of a purulent appearance; and it was remarked that in the latter case the quantity of the discharge was always more profuse. Although this disease be congenital, it may increase after birth beyond its original extent. Eight of the cases seen by Dr. Ascherson occurred in females, and three in males. These fistulæ now described have some analogy with the tracheal fistulæ recently discovered and explained by M. Dzondi; but the origin and the anatomical characters of the two are very different. In order that we may compare them, we have extracted the following remarks from Dzondi's narrative.

"At the anterior part of the neck, about the middle of the concave edge of the thyroid cartilage, there is found a small round opening, of about a line in diameter; its edges are neither red, tumefied, nor surrounded with any fleshy rim. It is not painful on being touched; and when firmly compressed, several drops of a puriform fluid may be made to flow out. A probe cannot be pushed very deep, in consequence of the winding track of the fistula, and on no occasion can it be introduced into the trachea, although a few bubbles of air almost always escape upon any forcible expiration."

These tracheal fistulæ may be associated, or occur in connexion with other congenital anomalies or irregularities of formation; especially with those which are denominated "monstrosities from asymphysis," that is, from an incomplete junction of the two lateral halves of the body. We cannot, however, take the same view of those described by

M. Ascherson, because they are not situated in the median line of the body; and as they have no communication with the air-passages, we must necessarily infer that their origin is not similar. He is of opinion that they should be regarded as proceeding from some anomaly or aberration of the nisus formations, congeneral with those which cause an arrest of the development of the fœtus, during one of those phases which it successively passes through, before it reaches its perfect state. To one of these transition forms belong the branchial fistulæ discovered by Rathke first in the young of the pig, horse, hen, water-snake, (coluber natrix,) and lizard, and afterwards in a human embryo, about 7 or 8 weeks old. These fistulæ or tubes consist in from six to eight apertures, arranged symmetrically on either side of the neck, opening into the pharynx, covered externally with a sort of operculum, and exhibiting on their inner surface several arched lamellæ. Rathke compares these apertures with the branchial apertures of the shark; and a beautiful confirmation of this opinion may be derived from the identity which exists in the vascular arrangements of fishes and of the early chick, as clearly made out and described by M. Huschke of Dresden. This anatomist publicly demonstrated that the aorta of the young chick gives off six branches, which pass on to the inner surface of the branchial arches, (or those lamellæ which are considered as rudimentary branchiæ,) and which afterwards communicate with, and terminate in, the descending aorta. Bäer has verified the existence of these branchial apertures in the fœtal dog and rabbit; and his observations have been confirmed by Burdach, Muller, Allan Thompson, and Becker. M. Rudolphi mentions having seen at Stralsund an infant, in whom the closing up of a fistula of this sort, brought on aphonia, epileptic convulsions, and other alarming symptoms, which gave way only when the ulcer was re-established, and the discharge permitted to flow. In one of the cases related by Dzondi, the healing of the fistulous opening was followed by a train of evils which finally proved fatal.

### II. Cases of Ague, treated with the Salicine.

Case 1. A youth, 14 years of age, after exposure to cold, and suffering from want of proper food, was seized with a tertian fever, whose attacks regularly came on at three o'clock in the afternoon. A vomit was first prescribed to clear the stomach; and then repeated doses of a mixture containing sal ammoniac and tartrate of antimony, for the purpose of acting on the bowels and of cleansing the tongue, which was loaded with a thick crust. The use of the salicine was then commenced, and 24 grains were given during the intermission; but the paroxysm returned, and the same dose was repeated during the second intermission: but as no perceptible effects had been obtained from it, recourse was had to the quinine, of which only eight grains were taken during the third intermission. The disease was checked from that time, and there was not afterwards even the slightest relapse of the fever; a circumstance which, it must be confessed, is by no means very common under any treatment. Whether any credit is due to the previous use of the salicine it is not easy to determine.

*Case* 2. An aged woman, who had long been a martyr to the gout, had suffered from three paroxysms of tertian fever, when Dr. Busch was called to her assistance. The cold stage was short, but the hot was tedious, and only towards the evening of the day, yielded to a gentle perspiration coming on at that time. During the intermissions, her health seemed moderately good; the use therefore of tonics was forthwith commenced. The cinchonine in doses of a grain and a half at a time was ordered, so that in all 15 grains were taken in 24 hours. The following paroxysm was not abated in severity; and the cold stage was even more violent, and the sweating was more copious than it had been heretofore. The same quantity of the medicine was repeated, and the fever now seemed to be checked; and this was really the case during the following twelve days, after which another paroxysm came on,

It is right to mention that the patient, contrary to advice, had discontinued the use of the cinchonine. Recourse was had this time to the salicine, of which five grains were administered every two hours, so that during the intermission forty grains in all were taken. No paroxysm came on, and the patient felt within herself quite comfortably, complaining only of a mist before her eyes. Although the medicine was not repeated, there was no relapse of the disease.

*Case* 3. A strong man, 52 years of age, who had suffered from tertian ague on the preceding year, was placed under the care of Dr. B. in consequence of a second attack. The cold stage was severe, the hot one moderately so, and the sweating was profuse. The tongue was coated with a thick yellow crust, and there were present the usual symptoms of a disordered stomach. A solution of the sal ammoniac and tartrate of antimony elicited copious evacuations upwards and downwards; and when the tongue became clean, four grains of the salicine were given every two hours: 36 grains were thus taken during the intermission. The following paroxysm was exceedingly mild, being marked only by a slight chill, and feelings of numbness in the limbs; but even these mild symptoms speedily ceased. The same quantity of the medicine was repeated only once more, and yet the patient remained quite free from any relapse.

*Case* 4. A man, 48 years of age, who had laboured under tertian fever in 1831, had another attack in the Spring of the following year, and was this time treated by Dr. Busch. After the primæ viæ had been well cleansed by vomits and aperients, the salicine was ordered in doses of five grains, so that eight doses were taken during the intermission. The expected paroxysm was arrested, but the patient complained of feeling much worse than he had done when the fever was allowed to come on; for his head was painful and confused, and strange figures and sparks of light kept passing before his eyes; and his wife reported, that he had been quite delirious during the night. In the morning he was very comfortable. The medicine was not however discontinued, and none of the disagreeable symptoms returned, until the afternoon when the paroxysm was again looked for;—then the cerebral disturbance was renewed, but it did not continue so long, nor were there any symptoms of pyrexia present, such as shivering, heat, or sweating. For other two days, the salicine was taken in the same doses as before; but then, contrary to all expectation, a severe paroxysm came on, and the shivering stage was remarked to be particularly violent. At this period the quantity of salicine which had been taken was 120 grains; and as this was deemed a fair trial, the quinine was substituted; it was ordered in doses of a grain and a half every three or four hours, during the intermission. From this time all threatenings of aguish symptoms entirely ceased.

Our readers will have no difficulty in drawing the legitimate conclusions from the preceding cases of genuine and well-marked ague, as to the comparative efficacy of the salicine and quinine. It may be useful to adduce an example or two, where the former medicine was used in the masked agues, or, as the foreigners term them "larvatæ intermittentes."

*Case* 5. A servant girl, 25 years of age, who for two years successively had suffered from very obstinate attacks of ague, consulted Dr. B. in the Summer of the year 1831,

Every second evening for some time past she was seized with violent head-ache, which continued with more or less severity during the night, terminating towards morning by a gentle perspiration. At no time was there any shivering or feeling of febrile heat, but the stomach was disordered, and the patient complained of general languor and uneasiness. An emetic and purgative were first ordered, and then the use of the salicine, in doses of two grains every four hours was commenced, and forthwith the head-ache entirely ceased.

I i 2

*Case* 6. A woman, 45 years of age, had had an attack of tertian fever in 1831. In the Summer of the following year, she suffered from a severe bilious disorder, and this left behind it a periodic stomach affection, marked by a sense of distressing pressure gradually amounting to pain, in the region of the stomach; this, after continuing for some hours, abated and ceased. Dr. B. suspecting that this was a case of masked ague, ordered the salicine; and its use was speedily followed by the complete removal of every symptom.

In addition to several other such cases as those now related, Dr. Busch alludes to the good effects which may be derived from salicine, in many forms of dyspepsia, in old catarrhs, when the bronchial discharge is very profuse, in fluor albus, and gleet, and also in some obstinate cases of hooping-cough; one particularly is mentioned of a child who had suffered from it for ten weeks, the salicine was ordered in doses of two grains repeated every three hours, and in the course of six days the little patient was perfectly ridded of all cough. —*Hufeland's Journal.*

---

### III. CASE IN WHICH THE POISON OF THE ROT (OF SHEEP) WAS COMMUNICATED TO A MAN.

A shepherd, 28 years of age, and of a healthy constitution, had been engaged in skinning the carcases of two diseased sheep, which had died of the rot, and in which the spleens were found much enlarged, easily friable, and of a blackish-brown colour. In a day or two afterwards, the man observed that several heat-bladders, (as he called them) which had existed for some time on his left fore-arm, had become more inflamed and painful, and he suspected that the blood of the diseased animals had infected them. In the course of the night he was restless and feverish, and could not sleep; his limbs ached as if he had been beaten all over, and then a shivering, succeeded by heats and intense headache, came on. Next morning the vesicles had become larger, and the whole arm was swollen, red, and painful; the pain was of a tearing and burning nature. Large linseed poultices were applied round the limb; but these had been on scarcely half an hour before the vesicles became quite black; and as they continued to increase in size, and the arm to become more painful, the hot poultices were exchanged for a sour dough or paste, to which about one-eighth of mustard flour and vinegar had been added. This application seemed to answer very well, for the pain speedily diminished, and the black gangrenous spots shewed no tendency to enlarge. The pulse at this time was 110, full and soft, the eyes were suffused, and the skin rather hot. On the inner side of the affected fore-arm, there were three gangrenous spots, in size varying from that of a penny to that of a sixpence; the arm was still much swollen even up to the axilla, the whole of the fore-arm was of a deep red colour, and from it there extended upwards to the shoulder, two broad red lines, which terminated above in enlarged axillary glands. Besides these local symptoms the breathing was tightened and oppressed, and the symptoms of general pyrexia continued. The mustard paste which had been applied to the fore-arm, as it probably kept up a good deal of irritation was now left off, and the unguentum elemi applied to the gangrenous spots. The constitutional treatment had consisted chiefly of cordial and antiseptic remedies; the diluted sulphuric acid was freely given in water as the common beverage; spirituous and ætherial medicines in small and repeated doses were frequently administered; and the action of the bowels was solicited by mild aperients. The eschars soon began to separate, and the tension and redness of the limb to subside; and now the dressings were made warmer by the addition of sulphur and volatile oils. In the course of eight or ten days he was so much recovered that he was able to resume his labours in the fields. There was a threatening of relapse, but by using proper measures in time, the progress of the mischief was arrested. The quinine and sulphuric acid fully re-established his health.

*Remarks.* It appears from the preceding, as well as from many other similar cases on record, that the action of most of the putrid animal poisons is decidedly depressing and of a septic tendency. The patient at first experiences great languor, and weakness in the limbs, headache, and a sensation of burning pain in the eyes, anxiety at the precordia, sickness and tension of the abdomen; and as these symptoms continue, every thing indicates a typhoid character. The correctness of this view is confirmed by the result of the remedies in the present case; we have seen that the relaxing poultices which were applied at first appeared to increase, rather than to abate the local mischief; whereas the stimulus of the mustard and vinegar was evidently serviceable. The utility of the internal remedies used leads to a similar conclusion. A strict antiphlogistic treatment in such cases, we consider to be decidedly injurious; we may indeed get rid of a portion of the unhealthy blood in this way, but we cannot correct its abnormal qualities; and no wonder, for it has become positively and essentially infected, and poisoned; and one great indication of cure must of a necessity be, to counteract the operation of the offending cause. — *Hufeland's Journal.*

Let our readers compare the last remark or two, with the reflections in M. Roche's Memoir, which is published in the present number.—Ed.

---

### IV. On the Use of the Sulphate of Cadmium against Specks of the Cornea.

Many years ago Graefe and Rosenbaum adduced several cases in which this new medicine had been used, with apparently good effects, in promoting the absorption of specks and opacities of the cornea. Dr. Tott of Mecklenburgh has lately repeated their experiments, and the results have been quite favourable to the report of his predecessors.

*Case* 1. A boy, 14 years of age, had a small spark of iron driven into his left eye; a violent inflammation of the cornea and conjunctiva was the consequence. Very active depletory measures were used, and when the inflammation was subdued, it was found that nearly one half of the cornea was rendered hazy and obscure by a blueish coloured opacity, which much impeded the sight. The ung. hyd., nitrico-oxyd. sulphate of zinc in the form of a collyrium, walnut oil, a perpetual blister behind the ear, were all tried without effect; the sulphate of cadmium was then used in the proportion of one grain of the salt to two ounces of water; 8 or 10 drops to be distilled into the affected eye thrice a day. In four weeks the opacity had vanished, and the vision was completely restored.

*Case* 2. A young girl had suffered from conjunctivitis and cornitis for six weeks, when she was brought to Dr. Tott.

A partial exudation of lymph into the texture of the cornea, had already taken place. The sulphate of cadmium was used in the same manner as we have described in the preceding case, and with equally satisfactory success.

*Case* 3. A young boy, affected with a scrofulous eruption on the face, and with inflammation of the left eye, was treated with antimonials, dulcamara, conium, and the result was the cure of both diseases. Unfortunately however a blueish white opacity remained on the cornea after almost all traces of ophthalmia had disappeared. Blisters behind the ears, zinc and lead collyria, with the internal use of mercurial and antimonial medicines were expected to effect the removal of the speck; but in vain. Many other remedies were then tried, but all fruitlessly, and at last Dr. Tott had recourse to the sulphate of cadmium; in eight days the opacity had sensibly diminished, and in five weeks more it had entirely vanished.

It is important to state, that the scrofulous affection of the face remained behind, so that we cannot well consider the malady of the eye as proceeding from the constitutional taint; at all events, the present case shews that the sulphate of cadmium cured it, although occurring in an unhealthy subject.—*Graefe's Journal.*

[The remedy proposed by our German brother deserves trial in this country.—ED.]

---

V. CASES ILLUSTRATIVE OF THE EFFICACY OF CONCENTRATED CHERRY LAUREL WATER IN NEURALGIA.

CASE 1. *Irregular Neuralgia of the Neck.*—A woman, 38 years of age, who had been labouring under violent meningitis (for which she was largely and repeatedly bled), was suddenly, during the decline of the disease, affected with a severe pain on the posterior and lateral parts of the neck; it returned at irregular intervals of time, but always with extreme violence; and the usual duration of each paroxysm was about two hours. The affected part did not exhibit any visible marks of disease, except a slight redness; but yet the gentlest pressure caused intolerable agony. Bleeding, leeches, acetate of morphia, taken internally and applied externally, hyosciamus with valerian and the oxyde of zinc, sinapisms to the feet, and a host of other remedies, had all been tried without effect.

Dr. Broglia was at this time first called to the patient; he ordered the pained part to be well wetted every second hour with a wash composed of three drachms of concentrated cherry laurel leaves water, and three ounces of rose water, the part to be kept constantly covered with a moistened compress. Almost immediate relief was experienced; and a complete cure obtained in three days.

CASE 2. *Femoro-popliteal Neuralgia.* A young female, after exposure to cold, when heated, was almost instantaneously seized with an agonizing pain in the knee-joint, extending for a few inches up the thigh and down the leg. The pain at first severe became gradually most excruciating, and after lasting for about two hours, it abated; only however to return at irregular periods, with its former violence. The general health was good. Dr. B. saw her on the third day after the seizure, and ordered a venesection and an active purgative as preliminaries. She experienced relief from these measures, but they did not prevent the return of the paroxysms; the internal use of sudorifics, turpentine, &c. and the operation of acupuncturation altogether failed; recourse was therefore had to the external application of the cherry laurel water, as in the preceding cases, and a complete cure followed in the course of a few days.

CASE 3. *Sciatic Neuralgia.*—A lady, during her convalescence from an inflammatory affection of the spinal cord, which required very active depletion, began to experience a severe pain about the right knee, darting upwards to the hip, and also down the leg. Friction, with different anodyne liniments, and the extract of henbane were employed without success. On the evening of the eighth day from the date of the seizure, the laurel water was first used, and its effects were speedily, and permanently also, fortunate.

The next case mentioned is that of a lady 32 years of age, who was suffering from severe irregular sub-orbital neuralgia, which had resisted leeching, quinine, belladonna, &c. but which quickly yielded to the laurel water wash.

The sixth case occurred in a young man who was labouring under irregular remittent neuralgia of the left scapula and shoulder. All the usual remedies, as iron, bark, colchicum, antispasmodics, acupuncturation, &c. having utterly failed, Dr. B. then used his wash, and we are told, with the happiest effects.

The Doctor is candid enough to admit, that the laurel water lotion has failed in several cases, which have been cured subsequently by blisters; and it is necessary to observe, that in scarcely any of the successful examples, had the disease existed long.

M. Roux published a paper in the Bullétin Génerale de Thérapeutique, for 1832, strongly recommending, in neuralgia, the external use of the following lotion.—Take of distilled cherry-laurel water four ounces—sulphuric æther, one ounce, and of extract of belladonna, two drachms. Mix.—*Annali Universali di Medicina.*

---

### VI. On the Development of the Thymus Gland in Man, and in other Mammiferous Animals.

There is no subject of physiological research more curious or interesting, than that of the mode and period of the formation of the different organs in living bodies. It was at one time believed that the "tout ensemble," or the whole, of the organized fabric was cotemporaneously and simultaneously evolved, during the time of the being's residence in its embryo and fœtal abode; but such is not the case, and it has been proved beyond all doubt, by the labours of some of the most eminent anatomists of the present day, that there is a regular succession of phases, or stages of development, and that in these different stages (which become gradually more and more complex), different organs of the body become first evolved. It is observed that some structures of the young being are peculiar to it, and become removed before birth ; such is the vesicula umbilicalis—others continue unchanged up to that time, and then undergo well-known changes, for example, the umbilical vessels, the heart, ductus arteriosus, and ductus venosus; while the rest are intended to retain their congenital characters, modified, indeed, by age, as long as life endures. Now it is to the second class mentioned that we may refer the thymus gland, for it seems to attain its full development anteriorly to many other organs, being largest and most perfect in very young animals, and destined speedily to lose its importance after early infancy. In the period of adolescence, when the body is supposed to have arrived at its mature formation, the thymus gland has shrunk in all its dimensions. The object of the present enquiry, is an endeavour to trace the different states or conditions which it exhibits at the different periods of life, and to determine what the epochs or periods are when the greatest changes take place. It must be noticed here, that the laws of the development and of the decrease of the thymus gland are not uniform and alike in the different tribes of the mammiferous animals. We shall, therefore, first try to ascertain these in the human subject, and then compare them with those which belong to other creatures, included in the same zoological class.

*Evolution of the Thymus in Man.*

The earliest appearance of the gland in the human embryo is about the tenth week of its existence. Behind the upper extremity of the sternum are to be seen two small bodies, which lie separate from each other, but are joined at the lower part by cellular tissue. They increase in bulk downwards, and almost equal the size of the lungs, although at this period of embryotic life, they do not acquire, compared with the volume of the whole body, a proportional enlargement so considerable as they do at a subsequent stage. [The lungs begin to be developed in the sixth week after impregnation, and the thyroid gland in the seventh.]

Cowper is, therefore, in error when he states—" in fœtu duorum a conceptu mensium, pro mole corporis major est thymus, quam in fœtu quinque, vel sex mensium"—*Anatomy of the Human Body, Leyden,* 1739; and the error is the more inexcusable, because no traces whatever of the gland have been discovered in a two-month embryo by Burdach, Meckel, or Wrisberg. In the third month the thymus is observed as a small body, situated at the base of the heart, and enveloped in a yellowish cellular substance; it consists of two granulations, placed on the sides of the trachea, and at this period, its relative size to that of the other thoracic viscera is the most diminutive. In the fourth month it acquires a considerable development, and extends from the base of the heart to above the region of the clavicles. The two lobes are still very apparent, and their granular texture may even be seen at this period. In the fifth month, the development of the gland is still greater, and its lateral halves become more closely joined. Wrisberg describes it in the following terms :—" It is composed of two principal lobes, and of a lobus succenturiatus ; the upper lobe (upper part of the lobe ?) lies over the left subclavian vein, with which it is loosely connected—the inferior lobe adheres to the pericardium, while the lobulus, which looks like a lymphatic ganglion, adheres to the right subclavian artery for the extent of about two lines. In the sixth month, the thymus sometimes rises as high as to reach the lower edge of the thyroid gland; but it has not yet been determined whether there is any juice or milky fluid contained in its substance at this time —in a seven-month fœtus, however, the author (Dr. Haugsted, of Copenhagen) was able to press out a few drops. The gland continues to increase in bulk up to the period of birth ; but its specific gravity diminishes in proportion as its cellular texture is unfolded, for, compared with that of water, it was found to be, in the eighth month, 1·099, and in the ninth to be only 1·071. The average weight of the gland, at the period of birth, is between two and three drachms. It has frequently been asserted, by anatomists of high repute, that the thymus, having attained its " summum" of development at the period of birth, begins from that time to decrease gradually, until almost every vestige of it is lost in advanced age. But more exact observations shew that the gland does not lose any thing of its volume in early infancy, and, on the contrary, that it rather goes on increasing for one or two years. True it is, that in some children, we may observe an appearance as if the gland had been compressed during the movements of respiration, and this may be really the case ; but still there is nothing to prevent a corresponding expansion backwards, and it will be found, on dissection, that this has generally happened. The changes which the thymus undergoes subsequently to the first or second years are of two kinds, and may be referred to two different periods. Up to the eighth or tenth year, a period which embraces the whole of the first dentition, and the early evolution of the permanent teeth, the gland appears to be nearly stationary, neither sensibly increasing nor contracting in bulk. Its cellular texture, however, becomes meanwhile more close, its milky juice less abundant, and, although still nourished by blood-vessels, the gland appears to exercise scarcely any function, and to have little or no influence on the rest of the system. Although its absolute weight remains all this time nearly unchanged, its specific weight sensibly diminishes. From 1·071, the specific gravity at birth, it was found by Dr. H. to have fallen, 14 days after birth, to 1·02· ; and, in a child 10 years of age, to be nearly as low as that of water. Its dimensions, however, experience little change, and almost quite agree with our description of them in the fœtus.

We come now to the consideration of the second period, dating that from the eruption of the permanent teeth. It is at this epoch that a striking diminution of the gland begins to take place; its blood-vessels shrink—its cells, hitherto perceptible, become obliterated ; it is, therefore, more solid, and less full of juice; its substance appears to be absorbed, and its lobes become thin, and more separate from each other. These changes are not very obvious till about the twelfth year, but after that, up to the sixteenth year, they are rapid and most easily traced. At this last-mentioned date, there are seldom any vestiges of the granular structure of the gland, nor of its division into lobes. Each of the lobes, separated from its fellow, has acquired the structure and colour of the thyroid gland, or rather of the spleen. The cellular substance which surrounds it, and which penetrates between the lobules, is now usually filled with fat. After the sixteenth year of age, the decrease and decay of the gland go on more slowly, but still gradually, until it becomes quite atrophied, and almost entirely obliterated. The atrophy and wasting proceed from below upwards; hence, in the adult, the remains are generally found immediately dorsad to the upper bone of the sternum. When its substance has altogether disappeared, there is found at this place a fatty mass, inclosing some brownish-coloured corpuscles—and these are the only traces of this organ. Meckel, Burdach, and Hewson are quite mistaken in their assertions, that the thymus is obliterated generally about the tenth or twelfth year. Dr. H. has found remains of it (imperfect indeed) at 21, and even at 28 years of age.—*Archives Gener. de Médécine.*

---

### VII. On the Impediments to easy Accouchement, from some Malformations of the Fœtus.

It has been too generally admitted, that those monstrosities only which are characterized by an excess or redundancy of parts, or by extreme malposition of these, can afford any real impediment to the expulsion of the child. A few examples will prove that this affirmation is not quite correct; but it will be proper, before particularizing them, to make an illustrative remark or two.

1. If the limbs of a fœtus be so wasted away, or so imperfectly formed, that they look rather like stumps, or like turtle-flappers, than like the ordinary lengthened extremities, the fœtus is necessarily more moveable in the liquor amnii, and must, in consequence of this, be more liable to a mal-presentation.

If, in such a case as this we have supposed, we should wish to deliver the child by turning, our diagnosis of the parts which we feel may very probably be obscure and perplexing—add to which, there may be no convenient part to lay hold of. It was in an example of this kind, that Pen was obliged to apply the crotchet upon the sacrum, and Delamotte confesses that he was once exceedingly puzzled to distinguish the different parts of a monstrous fœtus.

2. In anencephalous monsters, whose spine is found open behind, the head is usually thrown backwards, and the occiput is actually concreted with the cervical vertebræ. Such a malformation must seriously impede delivery (if the fœtus be moderately large), as the whole body is thereby an unpliant mass. Fortunately, these monsters are seldom carried the full time; but, even at the eighth month, they very commonly cause a tedious and painful accouchement. It is frequently necessary to effect the delivery by turning.

There are many other deformities of the fœtus, which may be unfavourable to the naturally easy expulsion of the child; such are adherences of the limbs to each other, or to the body, or of the fœtus to the secundines (an anomaly which is frequently associated with some malformations of the fœtus itself, as eventrations, &c. and which Geoffroy St. Hilaire considers to be, indeed, the cause of the malformations), or, lastly, an abnormal agglutination, or torsion of the navel-string. The mere death of the fœtus has been very generally supposed to render a labour less easy; and indeed the ancients believed that the expulsion was effected chiefly by the efforts of the child itself. To a certain extent, we must confess, this idea is correct; for there can be little question but that the death of the fœtus, by disturbing or arresting the utero-placental circulation, must have the effect of impairing the contractility of the womb, and should putrefaction have commenced, so as to render the flesh soft, loose, and inelastic, the fœtus must act almost as a plug, filling up the passage, and merely, perhaps, protruded by the violent contractions of the expelling organ, without any power of accommodating itself to the different turnings. When the body is in this soft state, it does not well transmit the impulsion communicated by the uterus to the head, but often rather bends or folds upon itself, and thus presents an unfavourable part to the os uteri—a part which is very rarely protruded first in a living child. The analysis of obstetrical tables will be found to corroborate this statement. Out of 15,652 births, at the Hospice de la Maternité, 689 children were still-born—of these 689, 539 were in a putrid state, or in the proportion of 7 to 9. The following exhibits the mode of delivery in these cases.

| | | |
|---|---|---|
| Born spontaneously | by the vertex / hips | 514 |
| | face.... | 3 |
| | shoulder | 4 |
| Delivered with the forceps.... | vertex.. | 5 |
| | forehead | 1 |
| Delivered by turning ........ | vertex.. | 7 |
| | shoulder | 3 |
| Delivered after craniotomy...... | | 2 |
| | | 539 |

According to this table, it appears that, in one case out of every 18 in which the fœtus was putrid, recourse had to be made to the use of the hand, or of instruments, to effect the delivery; whereas, in the labours of living children, the proportion is a great deal lower—once in about 60 cases.

To illustrate the remarks on the occasional impediments to natural accouchement, from some malformations of the fœtus, we shall adduce an example or two.

OBS. 1.—*Anencephalous Fœtus, born easily and naturally.*

A woman, 31 years of age, was admitted into the Maternity to be delivered of her fourth child. During her pregnancy, she had suffered much from cardialgia and anorexia; when admitted, the membranes had already broken and the waters freely discharged, but the labour-pains did not come on for a few hours afterwards. When the os uteri had dilated sufficiently, the presenting part of the fœtus could not be satisfactorily made out—it felt quite soft, and was at first supposed to be the genital organs, but the hips could not be traced; and, by continuing the examination, one of the arms was felt.

As the labour proceeded, the soft mass was protruded lower down, and in the middle of it, the accoucheur thought that he could feel a sort of opening, which received the extremity of the finger. The expulsion was gradually effected. On examination after delivery, it was discovered that the tumour was a large reddish fungosity, which covered the entire base of the cranium, and occupied the place of the brain. The fœtus was malformed in other respects, for the anus was imperforated, and there were no traces of external genital organs; there were also only four toes on each foot.

OBS. 2.—*Anencephalous Fœtus, presenting the Shoulder—Delivery by Turning.*

A middle-aged woman, pregnant of her second child, was seized with labour-pains in the eighth month. The pains had been preceded by hæmorrhage, and when the state of the parts was ascertained by "le toucher," it was found that the placenta adhered to the os uteri: higher up a hand could be felt, and, by following this, the fore-arm and arm might be traced; the fœtus was still alive, but very small—the delivery was effected by turning. The fœtus was monstrous, for the cranium was altogether destitute of its vault, and, in the place of the brain, nothing was to be seen but vesicular fungosities, full of serosity. It lived for a moment or two after its expulsion, but never breathed.

*General Remarks.* It is important to observe that, independently of the more frequent mal-position of a dead, than of a living fœtus, we have oftener the necessity of employing instruments in the one case than in the other; the foregoing table shews that this happens about once in 38 dead labours, even when the presentation is quite normal. If the data of other institutions agree with those furnished from the Maternité, the conclusion of the ancients, previously alluded to cannot be gainsayed; and, at all events, we must admit that a living fœtus is more favourable for easy expulsion than a dead one. But, in truth, the idea, although ridiculed by Petit, and by most subsequent accoucheurs, is not a whit more improbable than a favourite one of late years, we mean that which attributes the ordinary presentation of the head to an instinctive, and, in some degree, a voluntary effort or culbute of the fœtal being to direct its head foremost.

M. Paul Dubois read a very ingenious paper on this subject to the Royal Academy (of which an abstract is given in a late No. under the head of the Proceedings of the Institute,) but his reasonings are often fallacious, and many of the data on which they are founded are not correct. The great frequency of malpresentations in cases of deformed fœtuses cannot surely be attributed to the feebleness of the instinctive and spontaneous effort of the fœtuses, but depends much more probably on their excessive mobility in the liquor of the amnion, monsters being very generally smaller, and occupying less space than healthy children. Whenever the fœtus floats about freely in the uterus at the time of labour, the chances of malpresentation are much increased; and as it is only in the latter months of natural pregnancy that the fœtus occupies nearly the whole of the cavity of the uterus, and as the head is at this period heavier, although by a very small excess of preponderance, than the rest of the body, the reason of the most common, and therefore the natural presentation is sufficiently obvious. We do not however mean to deny in toto, the occurrence of any instinctive movements of the fœtus in utero, for we believe that they do take place, and moreover that their frequency is in proportion to the inconvenience and constraint of the position in which the fœtus chances to be placed; but these movements must be blind, and obedient much more to the operation of mere gravity than to the choice or the will of the young being. Within this limit we consider its instinct to be comprehended, that it endeavours to resist any painful or disadvantageous position, say, for example, the presentation of the face, (a presentation which is much more frequent when the child is dead, than when it is alive; the proportion being as 5 to 97;) and simply because certain muscles are thereby forcibly and distressingly extended; in consequence of this the living being struggles against it.

But surely this admission does not warrant us in saying that the fœtus instinctively chooses the position most favourable to its birth; with as much propriety we may suppose that it voluntarily turns round the occiput, so as to bring it under the symphysis pubis, during the second act of parturition; all that we can fairly infer is that it strives against any position or attitude which is painful or irksome. The ingenious doctrine of M. Dubois would therefore be more correct if it simply enounced "that the child contributes by its automatic movements, to the gliding of the head towards the most depending part, which is the cervix of the womb."—*Revue Medicale.*

---

### VIII. Torsion of the Arteries, employed in Amputation of the Leg, by Dr. Clot Bey.

An Arabian sailor was carried to the Marine Hospital at Alexandria, in consequence of a comminuted fracture of the left leg; the limb was amputated, and instead of using ligatures to the bleeding vessels, Clot Bey was anxious to make a trial of Amusat's proposal. He seized the arteries one by one, with a forceps, and drawing them out of their sheaths, he laid hold of them with the thumb and forefinger of his left hand above the grasp of the forceps; he then twisted them four or five times round upon their axes; and having done so replaced them. The stump was left exposed for a few minutes, in order that the operator might be satisfied, that the hæmorrhage was properly arrested; although there was no appearance of any, M. Clot confesses that he was by no means completely assured of the security of the practice which he had adopted. A tourniquet "d'attente" was therefore left around the limb. The wound was almost quite cicatrized by the fourteenth day; and there had not been once any threatening of hæmorrhage.

As far as success in one case entitles us to judge, the present must be considered highly favourable to Amusat's method.—*Ibid.*

---

### IX. Treatment of Fractured Limbs, by Inclosing them in Plaster Moulds, &c.

The eminent German surgeon Dieffenbach is in the habit of treating many cases of fracture, especially of the bones of the leg, by enveloping the limb, or only 3-4ths of its circumference, with plaster of Paris, after it has been ascertained that the fractured extremities lie in normal apposition with each other. It is of advantage to leave the anterior part of the limb exposed, as we have thereby an opportunity of watching the progress of the cure, and of applying any local remedies which may be necessary, especially when the case is one of compound fracture.

The principle of Dieffenbach's plan, that of an unmoveable apparatus round the bioken limb, has been adopted by many surgeons both of ancient and of modern times. It is to that glory of French military surgery, M. Larrey, that we are chiefly indebted for its establishment in present practice. In the Russian campaign, the method was most extensively adopted, and many a limb which had been fractured by gunshot injuries, and had been thus treated, was found on the return of the poor fellows to France, to have knit together most admirably, although the dressings had never once been removed from the period of the accident.

Larrey's apparatus has also the great advantage of being composed of materials the most simple, cheap, and almost always at command; for all that is required is a quantity of linen, straw, eggs, camphorated spirits, and of cold lead lotion. The mode of its adjustment is little more complicated than the application of a common bandage; and when once secured, the member is comparatively safe, and the patient, instead of being condemned to repose for a lengthened period, may be permitted to rise on the third or fourth day; and move about on crutches. Whatever be the limb which has been fractured, there is little risk of the apparatus being disturbed, even by a long journey; and if the jolting of the carriage be painful or improper, there is nothing to prevent the simultaneous employment of suspension, as proposed by M. Sauter. But often this is not necessary; for in from 24 to 36 hours, after the apparatus has been applied, it forms a compact, coherent, and accurately fitting mould or case round the whole of the limb, so that no one part of it can be moved without the rest of the limb following the motion; thus all danger of lateral displacement is securely guarded against. It may be supposed that it is less efficacious in counteracting the shortening of the broken limb than some methods of permanent extension; this may possibly be true, but when it is stated that, out of 8 cases of fractures of the femur, six were cured, without any shortening of the extremity; the argument cannot form any very potent objection to the practice; and indeed the very fact of all the muscles of the limb being equably and strongly compressed, (for the apparatus should be made to incase the whole of its length,) subdues in a great measure their tendency to contraction, and therefore obviates the tendency of the bones to ride over each other.

But if the immoveable apparatus which we have so strongly praised, and which our readers will find described at length in the 3d vol. of Baron Larrey's Clinique Chirurgicale, and also in an admirable thesis published lately by his son, possessed no other advantages besides the one of permitting the patient to move about safely during the treatment of his fracture, we should deem this sufficient to recommend it to the notice of surgeons; perhaps a case in point will be received as the most satisfactory evidence of the benefits accruing from its adoption.

A man of healthy constitution was admitted into a hospital for a complete fracture of the bones of the leg, about two inches above the malleoli. During six weeks he was treated by the common method, but at the end of that time it was found that union had not taken place. The foot being considerably everted, M. Berard first brought it into its right position and then retained it there by means of Dupuytren's splints, for another month and a half; and although there had been no displacement all this time, there was still a complete failure of any bony union. The immoveable apparatus was now applied; and when this had become sufficiently firm and compact, the man was allowed to get out of bed and walk about the ward on his crutches, without ever putting the broken limb to the ground. After the expiry of six weeks a perfect cure was obtained—*Archives Génerales.*

---

X. On the Progress of Physiological Science in the present Century.

The preface to the tenth French edition of Richerand's Physiology is written with much spirit by the eminent Baron, who avails himself of the opportunity to take a retrospective glance at the changes which physiological science has of late years undergone. "Since the first publication of this work, physiology has passed through a revolution which is every day becoming more and more complete. Towards the close of the 18th century the doctrine of vitalism reigned almost exclusively in our schools. Disciples of Borden, every thing was believed by us to be controled by, and subjected to, the supreme influence of organization and of the living principle; and the truths of physiology appeared to belong to a loftier and a more abstruse order than those of physics and chemistry. Influenced by the dictum of Aristotle, that where 'the natural philosopher stops, the physiologist begins,' we were unwilling to admit, but with the greatest reserve, the explanations afforded by pneumatic chemistry, which at that time was pursued with such brilliant success by men of the most distinguished talents. The remembrance of the injurious effects to medicine of the physico-chemical theories of Boerhaave and of Silvius, increased the distrust which discerning men had entertained against the explanation of living phenomena, by the laws operating upon dead matter. It was in vain that authors of the highest celebrity attempted to bring physiology under the empire of general physics, and to shew the similarity, if not the identity, of the forces which prevail in each. Every attempt was either repulsed at once without enquiry, or received with the most incredulous caution. Such was the spirit of the works of Pinel, and of his followers; of Bichat and of myself, at the period of the first publication of my physiology. Notwithstanding these obstacles, the fellow-labourers of Lavoisier, (and among these we need only name par excellence that most eminent of modern mathematicians M. Laplace,) persisted in their belief that physiology was only a branch of physics; and the then recent discoveries of Volta, of a new, a most active, and universally pervading agency, added a tower of strength to this side of the question. From the date of these discoveries, the attention of philosophers in every country was directed to trace and compare the curious and highly interesting relations of the phenomena of electricity and galvanism, in their ordinary and best known operations, with those exhibited by the nervous system of living animals. The important deduction is now established, that the instrument of the will and of the ideas, the nervous and cerebral system, differing according to the intelligence allotted to different animals, presents to our view diversities of conformation, of volume, of arrangement, and of proportions, as numerous as are the degrees of intelligence and of voluntary action. In addition to this, it has been ascertained that it is chiefly by the extension of the superficies, through the increase of the number of the convolutions, that the power of the nervous apparatus is exaggerated, just in the same manner as the power of a galvanic pile is exaggerated by adding to the number or size of the plates which we employ. Still it must be confessed, that we are very far from having discovered to what extent this important agent, viz. electricity, operates in the production of the vital phenomena, and that, what we do know of it, is much less than that which we do not know. In the functions of respiration and of digestion, it will be admitted by all to play a most important part, although we may be ignorant of the essential nature of these processes; and the day is perhaps not far off, when its relation with the phenomena of innervation will be made apparent, and all the mysteries of sensibility will, by its means, be explained. No physiologist has achieved more in this department than Dutrochet;—his work, wherein he develops the theory of endosmosis and exosmosis, may be considered as one of the most remarkable productions of modern times."

M. Richerand, having thus determined the character and objects of physiology, and having endeavoured to prove that at the present period the general aim in all quarters is to discover in the laws of physics the solution of the phenomena of organised matter, subjoins some remarks in explanation of the motives which have induced him to associate Professor Berard as a fellow labourer in the editing the new edition of his book.

"It is now several years," he says, "since I was engaged in researches relating to the condition of the organs in the embryo and fœtus, and which revealed to me that the human fœtus exhibits in succession all the forms, and follows all the degrees of organization and of life, so that before attaining to its perfect development, a development more exalted than that of any other creature, it has previously passed thro' the entire scale of animal existence. Instructed at the same time by the immortal author of 'The History of Fossil Remains,' (M. Cuvier,) that the debris of the animal kingdom which are buried in the antediluvian strata of the earth, display a progression of animals successively more and more complex in their structures, in proportion as these strata are placed nearer to the surface of the earth, and that the skeleton of man has never yet been found in a condition strictly and truly fossil—a fact which leads us to suppose that the Creating Power had called man into being, after the less perfect and less complicated animals had been formed, Impressed, I say, with all these considerations, I felt within myself as if I had ascertained the truth of one of the most general laws of organized matter."

But, not satisfied with the lofty pursuits of the philosophy of living beings, the Baron must soar a higher flight, and, penetrating the dark recesses of the metaphysical and political sciences, he strives to shew that there must be a gradation of moral feelings and of social rights and privileges, from the more simple up to the most complex, just in the same way as geology has shewn us that Nature has followed in the creation of organized matter.

The public may expect to be illumined on this mysterious subject, worthy of Kant himself, or of the most abstruse of transcendental philosophers, in a forthcoming work of M. Richerand, which, we are told, was interrupted by "that revolution, which, after having changed in a few days the destinies of our country, has extended its influence over all the nations of Europe, shaking political order, and threatening the very foundations of social economy!!"—Really we, on this side of the Channel, had no idea of the "hurly burly" effects of the "beautiful three days" of July.

Occupied with this grand topic, our author has deemed it right to avail himself of the aid of M. Berard, in making those additions and corrections which a new edition of his physiology required.

This preface of M. Richerand, although ingenious and interesting, is not likely to be assented to, in all its positions, by the intelligent English reader. Our French brethren are proverbially known to love change, and at the present moment their chief aim appears to be to bring physiology back to where it was under the reign of the mathematical, chemical, and mechanical philosophers of former days;—so true is the pungent remark of one of their own wits, who said, "There is nothing so new as that which is forgot."

---

### XI. ON THE DIAGNOSIS OF SOME SYMPTOMATIC AFFECTIONS OF THE HEART.

The diagnosis of diseases of the heart is far from being accurate and easy; and daily we meet with cases, which have been treated as such, but without success, recovering gradually of themselves, when the physician has ceased his officious interference

To distinguish between actual idiopathic disease, whether functional or organic, and the merely symptomatic and consecutive affections which may or may not occur during the course of other morbid states of the system, must therefore be an object of great importance. We well know that in nervous subjects, the slightest agitation of mind or body will at once induce many of the symptoms of cardiac disease; there may be the tendency to syncope, the fluttering, irregular, and intermittent pulse, the dyspnœa, and so forth, all present; but then the rapidity of the attack coming on, at least generally, after a fright or tale of distress, or a sudden surprise, and the gradual convalescence, under proper management, will soon dissipate our alarm. When such attacks as these recur frequently, and the system becomes, at the same time, more enfeebled and leuco-phlegmatic, the feet being œdematous, the face puffy, the breathing distressed, and the heart palpitating violently on any exertion, a medical man may be somewhat puzzled to decide, whether these symptoms are to be considered as indicative of heart disease, or of a systemic disorder, in which the heart, like other organs, is secondarily involved. If our patients be young, and especially if they be of the female sex we may, in a large majority of cases, suspect, à priori, that the latter supposition is the correct one. There are three morbid states of the system which, in particular, are often attended with symptoms simulating those of heart-disease, viz. chlorosis, anæmia, and incipient development of tubercles in some internal organ. The following case is an instructive one.

A female, 25 years of age, of a healthy constitution, and regular in her catamenia, had been treated during some years, at the Hôpital Beaujon, for severe palpitations and a breathlessness, which made her attendants apprehend the approach, or actual existence, of an aneurism of the heart. Under this impression, she was bled, leeched, and purged for several months; but no relief was obtained. The very opposite treatment, "inverse medication," was then employed, and she was rapidly cured, after she had been so much reduced by the Valsalva doctoring, that she had not strength to raise her arm from the bed. About the middle of last May, she was again distressed with her old complaint, which came on after a profuse menorrhagia. The palpitations and dyspnœa were so distressing, that the patient was obliged to keep her bed. She was again treated for disease of the heart by venesection and leeches, and to such an alarming state of exhaustion was she brought, that little hope was entertained of saving her life. Of this, however, her body appeared somewhat tenacious; for, by degrees, the immediately threatening symptoms abated, and she returned home.

M. Pigeaux was at this time called to attend her. On examining the region of the heart, he found the pulsations slow and feeble, but regular, subject only to occasional acceleration; no abnormal bruit was audible, although the second sound was sharper than in health, and resembled a good deal the first, or auricular one. The jugular veins were distended by the reflux of the blood, alternately with the impulsion of the point of the heart.

By percussion, it was easily ascertained that the size of this organ was of natural dimensions. Over the middle of the sternum, the sounds were obscured, and at the back they were scarcely perceptible. The respirations were much quickened, being from 40 to 42 in the minute. Mucous and sibilant râles might be heard over the whole of the chest, and there was some degree of dulness on percussion at the base of of the thorax, both in front and behind. The abdomen was very tender over the regions of the liver and of the colon, and also over the iliac fossæ. The pulse was soft, very compressible, and beat about 70 times in the minute. Every now and then the patient experienced attacks of syncope, which seemed to her to indicate the approach of death; and at other times she was so low, that she lay seemingly unconscious of every thing around her. The menorrhagia still continued, and her stools were found to be mixed with blood. M. Pigeaux, having carefully considered all the symptoms, especially those which were revealed by auscultation and percussion, and having ascertained, as far as he well could, the long previous history of the case, came to the conclusion that no actual and idiopathic disease of the heart existed; but so precarious seemed the state of the patient, that he gave a very guarded prognosis.—His attention was first directed to restrain the intestinal hæmorrhage; pills, consisting of two grains of dried extract of cinchona, and one of alum, were given every half hour. The hæmorrhage was stopped, but violent colicky pains came on, indicating, no doubt, the efforts of the bowels to evacuate the retained blood. These were gently assisted by the occasional use of mild lavements. The uterine discharge was next subdued, and the patient allowed a little wine, in addition to nutritious broths; the pulse began to rise, but the icteric symptoms (not mentioned before) were stationary. The sight and hearing, which at first were almost quite extinguished, regained a little of their activity, and the patient was now sensible of the questions which were addressed to her; by signs, she shewed that she understood their meaning. The alum in the pills was now exchanged for small doses of the subcarbonate of iron. This treatment was continued for a week and then the condition of the patient was so much improved, that M. Pigeaux deemed himself warranted in announcing his hopes of her ultimate recovery. The action of the heart became stronger, and at the same time more calm, and the pulsations of the jugular veins did not ascend above the middle of the neck; the second, or inferior sound had regained somewhat of its normal dulness. The patient lay constantly with the head very low, and she was cautioned against ever rising suddenly from the horizontal position. The sight had now so much improved, that she was able to distinguish between darkness and bright daylight. At the end of the second week, the icterus had almost quite disappeared—the speech was restored, and the appetite had become so vigorous, that she wished for more food than was expedient to allow her; but still the sight was very imperfect—it was only with difficulty that she could recognize a hand held before her. She was taking 10 grains of subcarbonate of iron three times a day; but this quantity was soon found to be too great, and the pills, consisting of the extractum cinchonæ and subcarbonate, were substituted. By the end of the third week, we are told that she began to shew "legers soins de propreté et de coquetterie," always a favourable sign with females, and soon after she had quite recovered.

*Reflections.* M. Pigeaux very properly condemns the ignorance, or extreme negligence, of this girl's previous medical attendants. The complete absence of all abnormal sounds of the heart, the feebleness, but regularity, of its pulsations and of those of the arteries, and the circumstance of the cardiac symptoms having been preceded and accompanied by a profuse loss of blood, ought, indeed, to have made evey one pause, before they subjected their patient to a course of treatment so painful, and so pernicious, as that which has been called by the name of Valsalva.—*Journ. Hebdom.*

Our readers will do well to re-peruse a paper by M. Bouillaud, on the Diseases of Young Females which simulate Disease of the Heart. It appeared in our last No. The converse of such cases, viz. where the anæmia and chlorosis are the effect, and not the cause, of the cardiac symptoms, is not unfrequently seen in practice, and mistakes in our diagnosis may be still more dangerous than in such an example as M. Pigeaux has afforded. We lately were called to attend a young lady, who had been treated by a physician for chlorosis, by large doses of subcarb. ferri, and in whom violent hæmoptysis came on. Disease of the heart had existed all the time.—ED.

### XIII. Of the newly-discovered Substance which has been called "Creosote," and of its Medical Properties.

This substance was found first in pyroligneous acid, and subsequently in all sorts of pitchy matters, by M. Reichenbach, during some experiments he was making on the products of the distillation of vegetable compounds. He gave the name of "Creosote" to it, from its property of preserving flesh from decay: (κρέας, caro; and σωζω, servare.) It is an oleaginous liquid, having the consistency of almond oil, is colourless, transparent, and possesses a high power of refracting light; its smell is penetrating, unpleasant, and somewhat like that of smoked meat; its taste is hot and exceedingly caustic; its spec. grav. is 1.037; it boils at 203° cent., and does not freeze at 27° c. The most interesting properties of this substance are the following. As soon as it is brought in contact with the white of an egg, the latter becomes densely coagulated; and so delicate a test is it of albumen, that if a single drop of the creosote be dropped into a very diluted solution of it, there is an immediate and copious formation of white pellicles. If a piece of fresh meat be steeped in a solution of creosote for half an hour, and then dried, it may be exposed for any time to the heat of the sun, without undergoing any decomposition; for, instead of becoming tainted, it becomes quite hard in the course of a week, assumes the smell of smoked meat, and a reddish-brown colour. Fish may be preserved in the same way. M. R. supposes that creosote is the preserving or antiseptic principle, possessed by vinegar, pitch, and smoke. When creosote is mixed with blood, the albumen of the serum is immediately coagulated; the coagula entangling and enveloping the colouring matter, while the fibrine appears to be unaffected: and it is probably in this way that it prevents and arrests the putrefaction of flesh; the mere muscular fibre, apart from the blood, having but little tendency under any circumstances to decay. When a drop of creosote is put on the skin, it speedily destroys the epidermis. Insects, fish, and plants are soon killed by being immersed in a solution of it. M. R. was led to believe that the medical properties of pitch, pyroligneous acid, the animal oil of dippel, and more particularly of the "l'eau empyreumatique," (lauded so highly by some in cases of cancer and of gangrene. It is obtained by adding common pyroligneous acid to a quantity of chalk until the effervescence cease, and then withdrawing by distillation rather more than one-half of the liquid,) might depend on the creosote contained in them; and he instituted therefore some experiments to ascertain its effects, in a concentrated and also in a diluted form;—but we have no satisfactory account of the results of these. M. Seiel has very accurately compared the solution in water of the creosote with the aqua binellii, and he is of opinion that creosote is the basis of that renowned styptic; and indeed, that this nostrum is only a very diluted solution of the creosote. M. Reichenbach employed the solution as a wash in some cases of burns, and with decided utility, not only in those without any abrasion of the surface, but also in such as had been followed by loss of substance and suppuration. The common itch, and several other cutaneous diseases also were benefited by its application. Carious ulcers were treated with advantage, by being well wetted with the creosote solution; toothache arising from decayed teeth was instantaneously relieved by a drop or two of the pure fluid instilled into the carious cavity; and even the mere gargling the mouth freely with the solution was often efficacious.

From this short statement it will be observed that the newly-discovered substance of creosote is nearly allied to many of the essential oils, and especially to the cajeput oil of medicine.

If the discovery of its presence in the famous Italian styptic, the Binellian water, and if, at the same time, the report of the virtues of this nostrum be confirmed, the art of surgery will have received a valuable boon from M. Reichenbach. The readers of our last

Number may, however, remember that, in the abstract of Baron Graefe's Report of the Surgical Hospital at Berlin, it is there stated that, in several experiments, the creosote solution, as well as the genuine water from Italy, had proved quite nugatory.—*Archives Générales.*

---

### XIII. ANATOMICAL NOTANDA.

### *Anatomy of the Eye.*

In a valuable monograph on the Anatomy of the Human Eye, Dr. Arnold, the celebrated author of the work on the Great Sympathetic Nerve, has entered into a minute description of each structure of this organ, as it was ascertained by accurate microscopical researches.

He confirms the statement of most preceding anatomists, that the cornea is to be viewed as a continuation, or prolongation, of the sclerotic coat, and that it is invested with a fine layer of the conjunctiva; this latter fact is best shewn by macerating the eyeball in hot water. We are told that the conjunctiva corneæ is of a serous nature, and that the conjunctiva scleroticæ forms, as it were, a transition-structure between mucous and serous tissues (?) The cornea itself is composed chiefly of lymphatic vessels, which Arnold could see distinctly with the microscope—the separation into lamellæ is altogether artificial. Between the sclerotic and the choroid is the arachnoid coat of the eye, adhering firmly to both of them —it is most easily detected in the eye of the fœtus. The choroid is to be considered analogous to the pia mater of the brain, and is no doubt, primarily, a prolongation from it, and, in the adult eye, retains an intimate communication with it by means of numerous blood-vessels, which accompany and surround the optic nerve. Its texture consists altogether of cellular substance and of minute vessels. The membrane of the aqueous humour is a shut cyst in the fœtal eye, as long as the membrana pupillaris exists, but becomes lacerated when that structure is removed. The iris is certainly not a continuation of the choroid; its texture appears to consist altogether of a vascular membrane, interlaced with numerous fibres, which are the extremities of the ciliary nerves. No trace of any muscular fibres can be discovered.—*Annales de Hecker.*

### *Observations on the Ganglion Oticum.*

Arnold having announced a few years ago the existence of a nervous ganglion, which supplied the auditory apparatus with the nerves of their organic life, and which might, therefore, be considered as analogous, in respect of the ear, to the ophthalmic or lenticular ganglion in respect of the eye, anatomists have very properly made numerous dissections, for the purpose of verifying or of disproving this statement. Among others, Stannius of Berlin, Muller of Bonn, and Boch of Leipsic, have published an account of their researches, which are quite unfavourable to the correctness of Arnold's supposed discovery; and more lately, M. Assman has brought out a monograph at Leipsic, in which he also attempts to prove that no such ganglion exists. The substance which has been described as a nervous ganglion, he says, is sometimes wanting altogether, and, when present, it appears to be a mere glandular-like mass, adhering to the sheath of the trigeminus. The nerves which Arnold has traced from the ganglion are, in truth, all derived from the adjoining nerves; for example, that one which, according to him, supplies the tensor tympani muscle, comes from the pterygoideal nerve, and his nervus petrosus superficialis minor is nothing but a shred of cellular tissue. The glandular nature of this supposed ganglion is very apparent in some of the larger animals, as the stag, and it has been generally observed to be smaller in old than in young animals of the same kind. M. Assman, in conclusion, has no hesitation in asserting, that the ganglion oticum Arnoldi is nothing but a lymphatic gland (and this is only occasionally present), and that what has been described as nerves, as proceeding from it, are merely filaments of dura mater or of cellular substance.—*Annales de Hecker.*

In reference to the subject of the preceding extract, it is but right to state, that several anatomists of high repute in Germany admit, in full, the correctness of Arnold's descriptions; and we find that, within the last month or two, Professor Mayo, of King's College, has completely satisfied himself of the existence of the ganglion oticum, although he had previously announced his failure in discovering it.—Ed.

---

### XIV. History of the last Illness of Goethe.

Almost every anecdote, however minute, respecting the life and dying moments of a man so loftily distinguished as the author of Faust, excites curiosity, and, if authentic, may often convey a useful lesson. To the medical man in particular, the study of human character is not only interesting, but of high importance for the right pursuit of his profession. The maladies of the body in patients so spiritual and intellectual as Goethe and Byron, are always more or less different, or are at least modified, from their ordinary features; for they seem to receive, so to speak, a colouring or hue from the character of the being in whom they occur. There is what may be called a "mental temperament" or "mental idiosyncrasy," just as there is a sanguineous temperament, or a lymphatic temperament, and so forth. Some may say that this is but a variety of the nervous temperament, and their assertion is true to a certain, but not to the full extent; every day we meet with numerous individuals, who may be truly said to have an extremely nervous constitution, one which is characterized by an exceedingly mobile or impressionable state of the nervous system, and in the treatment of whose disorders a special therapeutics is required, but yet who do not exhibit the features or character of what we have designated as the mental temperament; but these two may be, and very often are, associated in one individual; and such was the case, we should think, in Lord Byron, that marvellous compound of strength and weakness, of grandeur and littleness—a being, the restless activity of whose mind seemed to be too great a match for his corporeal energies, and whose body might aptly be compared to a harp chord, strung to the utmost, either vibrating to the fullest sweep, or snapping across on the simplest touch. Such a body, when affected with disease, must necessarily require a most delicate management; all over-excitement, or too great depression, will be found hazardous in the extreme, or, to pursue our simile, the harp-cord must ever be braced or unloosed with the softest hand and with the extremest gentleness.

It has been often remarked, that few men of distinguished literary talents bear vigorous depletion well; it seems to act upon them almost as a sudden refrigeration acts upon a heated glass vessel—the system has not the power of easily accommodating itself to the change of its condition, and its mechanism is fairly unhinged. Before pursuing our remarks any further, we shall briefly notice the most striking features of Goethe's last illness. This master poet of his age was distinguished no less by his wonderful natural endowments, than by the astonishing indefatigability of his intellect; for, not satisfied with having reached the proudest summit of poetical fame, his mind had discursively ranged over the sciences of botany, of philosophical anatomy, and of optics, and in each of these branches of science he has left monuments of his suprising acuteness. It was he who first pointed out the traces of an intermaxillary bone in man, and to him belongs the merit of having originated the idea, of a certain unity of formation being traceable throughout all animals, which Geoffroy St. Hilaire has subsequently so beautifully unfolded and illustrated. In vegetable physiology, he shewed by his work on the Metamorphosis of Plants, that he was at least a quarter of a century before its then existing condition. Whatever, in short, he applied his mind to, received the impress of its glowing activities.

The exterior of Goethe was in harmony with the fine spirit which was tenanted within; he was tall, well proportioned; his chest was large and dilated, his limbs strong, his sensual organs sound and active. In youth he had frequently indulged to excess in the gifts of Bacchus; but in his later years he was exceedingly abstemious, at least with respect to drinking. He had the peculiar idiosyncrasy of being affected by unusually small doses of medicine; a tea spoonful of tincture of rhubarb, acted as a moderate aperient on his bowels, and a quarter of an oz. of Glauber's salts operated with drastic severity. His constitution, although naturally fine and vigorous, had suffered after the completion of almost every one of his great works; the outlay of mental strength seeming to be uniformly followed by an exhaustion of his bodily energies.

Towards the close of the year 1830, Goethe had a severe attack of hæmoptysis; but from this illness he recovered completely. On the 16th of March, 1832, he had gone out in a carriage to take an airing, and on his return home he began to complain of having suffered from the chill air; his appetite failed, which was not usual with him; and feeling generally indisposed he retired early to bed; but his sleep was disturbed by a troublesome dry cough, and by repeated chills followed by short fits of feverish heat. On the following morning his countenance was dull, his breathing distressed, but there was no pain of the side; he was thirsty and much annoyed by eructations of large quantities of flatus from the stomach; the abdomen was tympanitic, rather tender on pressure, and the bowels had not been relieved for two days. The skin was dry, urine turbid, pulse moderately full; he said that he felt as if his head was empty, and that he could not combine two ideas together; his deafness (with this he had been troubled for a considerable time previous) had increased; and he was constantly repeating a favourite apophthegm of his own; "When a person has no longer a right to live, he must be contented to live as he can." A gentle aperient and mucilaginous drinks were prescribed; when the bowels were relieved he expressed that his general feelings were more comfortable. Small doses of the red sulphuret of antimony were ordered to be taken at short intervals. The distinguished sufferer was so much benefited by these means, that in the course of two or three days his friends thought that he was quite convalescent. But, alas! these hopes were to be blasted; during the night of the 19th he had a return of the cold shiverings, and these were accompanied with startings of the limbs, and with much precordial anxiety. On the following day, Dr. Vogel, the physician of the Grand Duke of Weimar, was summoned to his assistance; he found him in a state of extreme agitation, tossing about from one side of the bed to the other, for he could not find any position that was comfortable; his teeth chattered together; his looks were much altered, the eyes were sunk, and every now and then he complained of sharp pains in his chest. There was a cold clammy perspiration on the skin, the pulse was quickened and hard, but could not be very distinctly felt; the abdomen was tympanitic, and his thirst ardent. He himself dreaded a return of the hæmoptysis.

An infusion of peppermint and chamomile, to which some æther and solution of anisated ammoniacum were added, was ordered to be administered at short intervals; the heat of the surface then returned in a short time, and the pain being fixed over the great pectoral muscle, a blister was applied there. In the evening he felt himself better, and his physician found him seated in his arm chair, from which he never rose afterwards. On the 21st the unfavourable symptoms were decidedly aggravated; the prostration of strength was extreme, and occasionally the mind was quite bewildered and delirious. A loud sonorous rale in the chest was distinctly audible; the lips were glued together with a viscid mucus, the tongue was coated with the same; the expression of his features at this time was however calm; and this appearance alone sufficiently attested that the patient did not suffer much bodily distress; for, with his constitutional impatience, it would not have been long concealed. From several expressions which dropped from him, it was quite manifest to the attendants, that Goethe was not at all aware of the nearness of his dissolution. His speech gradually became more and more confused; "more light," (mehr licht) were the last words which he spoke; and when the tongue refused any longer to utter his ideas, he would endeavour to express them by signs; at first in the air, and then, as his strength failed more and more, on the bed-clothes.

At half past eleven he expired, resting on the left corner of his chair.—*Journal de Hufeland.*

The preceding report is instructive, as far as it goes; for without a post-mortem examination, it is almost impossible to arrive at a very accurate diagnosis of the real nature or the seat of Goethe's illness; the impression left upon our minds from an attentive perusal of Dr. Vogel's account is, that the disease was a masked or indistinct bronchitis; and if our judgment be correct, it must necessarily follow, that we altogether disapprove of the treatment adopted by the medical attendants. The Broussais plan is the one which we most certainly should have recommended; no large bleedings, but only the carefully repeated application of leeches; and in addition to this, we should have prescribed refrigerants, such as the nitrate of potass, the acetate of the same, alcali, the spiritus mindereri, or such like.

But it is unnecessary to enlarge on this hypothetical case, and our only motive in so far expressing our opinion is, to encourage our readers to analyse for themselves many of the reports of medical cases with which every journal teems. It is a useful exercise, and is well calculated to train the mind to accurate reasoning.

We regard Goethe's last illness as a very faithful pattern or sketch of disease, as it often exhibits itself in many patients of high mental endowments: there is an irritability or fretfulness of the system, associated with a condition of bodily energy, '*below par*;' and it is this compound state of the machine that we should ever keep in view, when called to treat its disorders. We have often had occasion, in practice, to remark an analogous state of body in patients who were a prey at the time to any depressing mental emotion, for example, in bankrupts, in gamblers, prostitutes, and in those who were secretly pining from neglect, or disappointed hopes.—Rev.

### XV. Calcareous Incrustation on the Crystalline Lens.

This incrustation occurred on the lens of a horse, covering nearly the whole of its anterior surface; it was hard, friable, and irregular or slightly mammillated; of a yellowish-white colour, and weighed rather more than eight grains. The texture of the adjacent lens had become softened, and almost puriform, so that mere washing separated the concretion from its attachments. M. Lassaigne, on analysis, found that it consisted of—

| | Parts. |
|---|---|
| Albuminous Animal Matter.. | 29.3 |
| Phosphate of Lime ........ | 51.4 |
| Carbonate of Lime ........ | 1.6 |
| Alcaline Salts.............. | 17.7 |
| | 100. |

It may be regarded therefore as a sort of ossification, containing however an unusually large amount of the earthy phosphate.—*Journ. de Chimie Medicale.*

### XVI. Muller's Experiments on the Blood: the Fibrine supposed to be in a State of actual Solution during Life.

The common opinion, as to the manner in which the coagulation of the blood is effected, is, that the clot or crassamentum is formed, and separates from the serum, by the aggregation of the red globules, which are supposed to be fibrinous corpuscles, invested with a layer of colouring matter. When the clot is washed with water, so as to become colourless, it is imagined that the colouring matter only has been detached, and that the pure fibrine, which, according to this opinion, had hitherto existed in the clot as distinct granules or nuclei, now remains agglutinated together into one mass. Sir E. Home, MM. Prevost, Dumas, Dutrochet, and others, have taken this view of coagulation. Berzelius, however, reasoning from the circumstance, that lymph contains a certain portion of fibrine in solution, has conjectured that the fibrine of the blood may be virtually and truly dissolved in the circulating mass during life—that the serum is to be considered as a fluid strained off from the blood, and that the clot is formed by the precipitation of the dissolved fibrine, which, as it separates in the solid form, entangles and agglutinates together the red globules. To determine this question, Muller has performed numerous experiments, and the result of these has been to satisfy himself of the correctness of Berzelius's conjecture, that the fibrine of the blood is actually in a state of solution during life. If some frog's blood (the globules of which are proportionally very large) be received into a watch-glass, we shall find, by examination with the microscope, that before the coagulation is finished, a few colourless, almost transparent, coagula are formed; these may be lifted up and separated at the edges with the point of a needle, and if the blood is poured off in a minute or two after it has been discharged, we may observe several points and small pieces of this colourless coagulum adhering to the bottom of the watch-glass.

These appearances Muller has repeatedly witnessed. If the blood be previously diluted with a little serum, so that the red globules are more sparse, and kept farther apart from each other, these minute colourless coagula may be seen in the intervals between the globules; and, indeed, it appeared as if the scattered globules were held together by these coagula; for it was observed that they were all dragged along together, and at the same time, by merely tearing with the needle's point the intervening coagula.

The blood of the frog is much better fitted to exhibit these phenomena than human blood, because the globules in the former are proportionally so much larger than in the latter. There is another, and a still more convincing method of ascertaining that the fibrine is really and truly dissolved in the living blood:—Let a small quantity of frog's blood, previously diluted with an equal quantity of water, be filtered through common filtering paper, the globules, being large, do not pass through, and an almost colourless serum (slightly tinged red, from the presence of a minute proportion of the colouring matter which has been dissolved by the water) is obtained. If, instead of adding pure water, we use a weak solution of sugar, the filtered serum will be quite colourless, and free from any admixture. When this serum is examined with the microscope, no traces of any globules can be discovered; but in the course of a few minutes, an almost perfectly transparent coagulum becomes perceptible, and this may be drawn out on the point of a needle. If allowed to remain, this coagulum thickens, becomes opaque and fibrous, and resembles much the coagulum of human lymph. In this way, the fibrine of the blood may be obtained in its purest state. It is to be remembered, that only a minute portion of it indeed is dissolved in the filtered serum, the larger portion being detained on the paper, in consequence of it becoming coagulated before it has time to pass through. If the serum, as it filters, be received into a watch-glass, in which there is some liquor potassæ, numerous flocculi are gradually formed; this appearance is still more distinct, when sulphuric æther is used instead of the liquor potassæ. It is of importance to remember, that the albumen of the serum is not affected by either of these agents.

The liquor ammoniæ, acetic acid, solution of muriate of soda, or of subcarbonate of potass, do not produce any visible effects on the filtered serum. The best method of ascertaining the actual quantity of fibrine in blood, is by well whisking and stirring it about. In this way, the previously dissolved fibrine may be procured as an almost colourless mass, while the globules remain suspended in the serum; and these will be found to have retained their natural appearance, provided no water has been added. Berzelius most erroneously asserts, that after the fibrinous matter has been completely separated by agitation, no entire or perfect globules can be discovered floating in the serum, but only small broken corpuscles, which he regards as portions of the colouring capsules of the globules. They, indeed, will pass through filtering paper; but it is to be remembered that the entire globules of the blood, in the higher animals, will always pass through.

All the experiments which Muller has performed have satisfied him, that the globules of blood, from which the fibrinous portion has been separated by stirring, remain unchanged, and of their natural form and size, for several days; that even their flattened discs remain distinctly perceptible, and that they continue to float in the serum, near its surface, and do not sink to the bottom, as Berzelius has stated. This last circumstance indicates the high relative specific gravity of the serum in the more perfect animals. In a mixture of the serum and red globules of frog's blood, the latter are quickly precipitated; and the same is true of mammal blood, if water be added to it; part of the colouring matter is dissolved, and part is thrown down to the bottom of the vessel.

The important conclusion which Muller wishes to impress upon his readers is, that the fibrinous part of the blood is in a state of actual solution during life. Whether the nuclei of the red globules consist altogether, or partly, of fibrine, he has not yet been able to determine.—*Burdach's Physiologie als Erfahrungswissenschaft.*

---

### XVII.

### Institute of France.

### I. Academy of Medicine.

*Sittings in October.*

Induction of Premature Labour. M. Stroltz, member of the Faculty at Strasbourg, communicated the particulars of a case in which he induced labour at the end of the seventh month of pregnancy, in a woman, 29 years of age, hunch-backed, and otherwise deformed, and who had been delivered in her two preceding labours by the operation of embryotomy. He introduced pieces of prepared sponge into the neck of the womb; as these became moistened with the discharge of the part, they gradually dilated the passage, and at the end of three days, the woman gave birth to a living child. It lived for three months and a half; and the mother survived eight months after her accouchement; the cause of her death was pulmonary consumption. On examining the body after death, it was ascertained beyond doubt, that the pelvis was too much contracted, ever to have given passage to an ordinary sized fœtus of nine months.

[We are not informed of the opinion of the Academy on the preceding communication, but the Editors of the Revue Medicale, from which the report is derived, have subjoined some remarks of their own, and as they express the sentiments of by far the larger portion of the French medical school, it may be well to subjoin them.

"The practice of M. Stroltz is certainly very bold; but we cannot take upon ourselves to recommend its imitation.

To provoke labour before the appointed and natural time of occurrence, is to assume the power of condemning to death a living being, or at least of greatly diminishing its chances of existence. Now, can a medical man, with the approval of his conscience, pronounce upon the propriety, and can he be the direct author of such a step? Is he permitted in any way to compromise, if not to sacrifice, the life of the child for the safety of the mother?

These are delicate questions, which involve alike many moral, religious and political considerations; and surely they ought never to be boldly solved by any physician alone. While any doubt exists, it will be well to abstain from such a hazardous attempt.

The practice of inducing premature labour has indeed been frequently commended and practised by English and German accoucheurs; and we are told that, in addition to the advantages to the mother, when it has been ascertained that her pelvis is not capacious enough to permit the exit of a full sized child, numerous statistical or tabular reports of midwifery writers, sufficiently shew, that artificial premature labour, say at the seventh month, is not at all more dangerous to the life of the child, than the Cæsarian operation, or indeed than most of the manœuvres we are compelled to adopt, in difficult and protracted labours. But can we fairly and conscientiously deduce very exact inferences from all these reports, when we reflect upon the marvellous resources, (and these too often altogether unexpected) of nature to expedite and complete many accouchements which had been pronounced almost impracticable by good and expert physicians?"]

### Cowpox, origin of.

The report of some experiments lately performed by M. Fiard, was read to the Academy. Jenner had announced that the cow-pox was a disease generated by the inoculation of the matter of the "grease," or as the French term it, "eaux aux jambes," in horses. Some physicians, on the other hand, viewed it as an idiopathic, and not as a consecutive disease, and were of opinion that it occurred primarily and spontaneously in the body of the cow, just in the same manner as the rot is a disease peculiar to the sheep; and lastly Dr. Robert of Marseilles adopted the hypothesis, that cowpox is nothing else but the small-pox, communicated to the cow, and modified, and rendered more mild by the peculiarities of the constitution of the beast. With the hope of throwing some light on this very interesting, but obscure subject, M. Fiard instituted a variety of experiments. On the one hand, he inoculated four cows with the virus taken from "greasy" horses; and on the other he inoculated eleven cows with the matter of genuine variolous pustules, occurring in the human body; but in neither set of cases was any disease communicated. M. Fiard then was anxious to repeat the experiments of Dr. Sonderland of Bremen; [these experiments, as most of our readers probably know, consisted in enveloping cows with the bed-sheets and coverings which had been used by variolous patients, and which were therefore impregnated with the cutaneous discharge.] But as they had been very carefully tried without success at Alfort, he thought it unnecessary to go over the same ground. On the whole he concludes from his researches, that the vaccinia is a disease truly and purely "vaccal," or peculiar to the constitution of the cow, and not the result of any preceding inoculation. M. Girardin confirmed the statement of M. Fiard, that the experiments recently instituted at Alfort, for the purpose of ascertaining the accuracy of Dr. Sonderland's conclusions, were most unfavourable, and indeed quite opposed to them. In vain cows were kept covered for several days with the bed-sheets, &c. of variolous patients. No results ever followed, indicating the transmission of any disease: and as the experiments which have been performed in England and Italy also, for the same purpose, have been equally nugatory, we are bound to discredit all the premises on which Dr. S. has founded his theory, of the identity of the variolous and vaccine poisons.

M. Huzard stated that the old vaccination committee had repeatedly attempted to produce vaccinia, by inoculating cows both with the matter of the "grease," and with that of the human cowpox vesicle, but always unsuccessfully.

M. Boiveau Laffecteur alluded to the cases of himself and of his child, both of whom had been vaccinated, quite successfully, with virus taken directly from the cow. This inoculation was repeated on the cow, but it did not succeed.

[In another report of the Academy-proceedings, we find that it is sated, on the authority of M. L. that the genuine cow-pox has been induced in the cow, by inoculation with the virus of the human vesicle.—Ed.]

Mr. Salmade confirmed the statements of M. L. respecting the mutual communicability of the vaccal virus to man, and of the human vaccine virus to the cow.

### Treatment of the Cholera.

Dr. Petit, physician of the Hôtel Dieu, read a memoir on the means best adapted to induce re-action in the cold stage of cholera. He alluded to his practice during the preceding year, of applying along the whole length of the spinal column compresses, wet with a mixture of equal parts of liquid ammonia and spirit of turpentine, and of then covering these compresses with others, which had been wrung out of boiling water, and, lastly, of passing frequently along these last a heated iron, for the purpose of keeping up a constant evolution of the stimulating vapour in contact with the skin. Although satisfied, then of the advantages to be derived from this procedure, he found that several inconveniences attended its adoption; for example, it required that the patient lay on his belly, and, moreover, it was not easy to keep the rest of his body covered and warm at the time. With the view of avoiding these inconveniences, M. P. had used more lately an apparatus, which was at once simple and quite efficacious. It consisted of a tin box, 2 feet long, 8 inches wide, and $2\frac{1}{2}$ high, and this was received into the centre of a cushion, filled with bran or oat-chaff, the edges of the hollow in the cushion turning over and covering the angles of the box. The box is to be filled with boiling water, in which a quantity of common salt has been dissolved. The back of the patient being applied over the box, is thus exposed to a constant and steady heat, which evaporates the stimulating fluid of the compresses; and this local excitement, if kept up for one half or three quarters of an hour, Dr. Petit has found of most decided utility in restoring the circulation, and in rousing the nervous system.

Conjoined with this local treatment, he recommends the internal use of light aromatics and antispasmodics, and allows the patient to suck small pieces of ice. Out of 14 cases of cholera, some, too, having already reached the cold stage, the above practice was successful in completely recovering 13.

### Nutrition and Diseases of the Humours of the Eye.

M. Professor Bouillaud, in the name of a commission, read a report on the memoir of Dr. Bourjot St. Hilaire, on the explanation of the nutrition, and of some diseases, of the ophthalmic humours, by the laws of endosmosis and exosmosis. The memoir is divided into three sections—the first is devoted to the consideration of the various theories which have been advanced, at different times, to account for the formation and renewal of these humours; and alludes to the opinion of M. Ribes, that the aqueous humour is secreted by the ciliary processes—to that of M. Petit, that the lens is nourished by a simple imbibition—and to the doctrine of M. Blainville, who regards the lens as an unorganized structure, and who rejects, therefore, altogether the supposition of its being vascular, and of its opacities being in any measure connected with inflammatory depositions. In the second section of the memoir, the reader's attention is directed to the beautiful explanation which Dutrochet's theory affords, of the formation and renewal of the ophthalmic humours. Whenever there are two fluids, of different densities, separated only by a thin membrane, a strong movement of endosmosis, of the less dense fluid, is immediately established, towards that one which is more dense, and, at the same time, a light current of the denser to the less dense fluid takes place, so that an equilibrium of density is effected, or, at least, a tendency to such an equilibrium is begun. Now, it is in such a passing and repassing, that the mechanism of the circulation of the ophthalmic humours consists, according to Dr. St. Hilaire: thus, he supposes that the aqueous humour passes, by endosmosis, through the crystalline capsule, and in the same manner the liquor Morgagni may pass into the cells of the vitreous body; and this last may possibly be transmitted into the venous vessels which are distributed on the surface of the hyaloid membrane—the different densities of the three humours, separated as these are from each other, only by very delicate partitions, giving rise to constant endosmotic and exosmotic actions. In the third section, the author endeavours to trace the origin of some diseases, such as cataracts, to the operation of these agencies; the fluids he supposes to vary occasionally in their respective densities, and, consequently, in their reciprocal transmissions. But this speculation, although ingenious, is not founded in any observed data. The Commission, however, in conclusion, awarded their high approval to the memoir of Dr. St. Hilaire.

*Sittings in November.*

### Suture of the Perineum.

M. Roux directed the attention of the Academy to the very frequent failure of all attempts to unite lacerated wounds of the perineum, by means of the twisted suture. Of late, he himself has quite abandoned the use of this suture, and has substituted the quilled suture, with much more satisfactory results. About a year ago he operated upon a female, who has since given birth safely to a child, and he has more recently cured another woman, who had laboured under the disease for the space of two years; he also alluded to two additional cases, which had terminated equally well by means of the quilled suture. In most of these cases, the threads were withdrawn on the 7th day. It is always necessary to caution our patients against any straining at stool, and that they should keep their limbs close together. M. Roux does not approve of M. Dieffenbach's advice, to make one or two lateral incisions when we employ the suture to such wounds. The practice not only adds to the pain, but is often positively injurious in its effects, by the contraction of the cicatrices.

### Epizootics.

M. Dupuy read a report upon a memoir of M. Fodéré, respecting an epizootic disease which prevailed, in the years 1821 and 22, through the department of the Bas-Rhin. The affection was seated chiefly in the lungs, and its characters assimilated it to the nature of scirrhus and tubercles. M. Fodéré deemed it to be truly contagious; but his reporter is inclined to believe that it was rather endemic and hereditary, for traces of its effects were discovered in the lungs of the unborn fœtus; and what confirms him in this view of the case is, that M. Fodéré himself recommends certain hygienic measures as prophylactic of the disease. Generally speaking, all such measures fail in arresting any epizootic; and it is much better to kill immediately all the infected animals—the main object being less to cure, than to ameliorate and renew the breed of animals.

If the disease were epidemic, such an Alexandrian method of solving the knot would be quite unnecessary. M. Fodéré had proposed that no meat should be sold at market, without a certificate of the soundness of the animals slaughtered; and, moreover, that all infected animals should be forthwith buried entire and unskinned. But both of these precautionary suggestions seem unnecessary; and, indeed, the first one must inevitably prove a great hindrance to trade, and the latter would cause a very considerable loss to the public. M. D. is of opinion that the skins seldom, if ever, propagate the disease; and he doubts that even the flesh of an infected animal does so.

To procure good stocks, to cross the breeds, and to feed well—these are the only true ways of improving our domestic animals; and it has been by attending to these particulars, that the English have succeeded so admirably in bettering their butcher-meat, both in respect of quality and of size, the weight of the oxen being raised, we are assured, from 200 to 400 kilogrammes.

The chief object to be pursued, is to couple such males and females as have a large development of the soft parts, and a small development of the bones, and this crossing must be repeated very frequently.

### Medical Responsibility.

M. Velpeau, in the name of a commission, reported upon the following case.

A young woman was safely delivered of her second child, and every thing went on well until the fifth day, when fever set in, and the patient died seven days afterwards. The uterus and vagina were found inflamed, the substance of the former was friable, and its inner surface was covered with granulations, of the size of peas. The accoucheur was accused of having hurried the labour, and of having left a portion of the placenta behind, by several medical men, who signed a "procès-verbal," in which the several charges were stated.

An appeal was made to the Academy by the accused, and the commission appointed to investigate the matter now declare—1, that it is their opinion that no portion of the placenta was left behind; 2, that even had there been any remaining, the accoucheur is not therefore necessarily to be blamed, and, 3, that the conduct of those who preferred the charges is improper and censurable.

Some of the members of the Academy were of opinion, that the last declaration ought to be suppressed.

---

### II. Academy of Sciences.

*Sittings in October.*

### Physical and Therapeutical Properties of Chromate of Potass.

M. Jacobson, a foreign associate of the Academy, communicated the results of a very extended examination of this substance. It sustains, without decomposition, a very elevated temperature, unless carbon be present, and then a change is readily induced, at least under certain conditions, and this change is always accompanied with a very bright incaudescence. This property may be turned to a very useful account, in promoting the combustibility of many vegetable substances, as linen, cotton, paper, &c.; for it is only necessary to wet them with a solution of the chromate, and, when dried, they will burn with much heat and light, but with no flame. M. J. is of opinion, that not only is the chromic acid decomposed during the combustion, but the potassa also, by means of the carbon and chrome together. Among the practical applications which may be made of this property of the salt, our attention is directed first to the preparation of moxas. We have only to wet a sheet of paper, previously rolled into the proper shape and size, well with a solution (1 part to 16 of water), and our moxa is made. It will burn completely away without any fanning. The chromate is admirably adapted to the composition of pastilles; but its main importance is, that with its assistance matches may be quickly made for the artillery. Another property of the chromate of potassa (that of being able to combine with different animal and vegetable substances, without suffering decomposition) renders it of use in preventing, in a multitude of cases, the processes of fermentation and of putrefaction; but, for this purpose, the bichromate is better suited than the neutral chromate. A very dilute solution (4 parts to 1000 of water) will be found to preserve anatomical preparations, without changing their form or consistence; and it has, moreover, this advantage, that it does not oxidate the steel instruments which we may be using. M. J. assures us that, after having been kept in the solution for fourteen months, he has seen preparations taken out, which might be dissected as well and easily as if they had been quite fresh. It is right to state, that all parts are not equally well preserved; for example, nervous substance becomes much altered—membranous structures are the best adapted.

Applied externally, the chromate acts as a discutient, and a very concentrated solution will cause corrosion of the part. It has been used successfully against some sorts of ulcer and of cutaneous disease.

Given internally, it produces vomiting as speedily as the tartrate of antimony; in the dose of half a grain every two or three hours it induces nausea, and may, therefore, be employed advantageously in some pectoral complaints.

### Comparative Number of Male and Female Children—the Causes, &c.

M. Girou, the author of a memoir on this subject, had previously directed the attention of the Institute to the probable causes which may determine, or at least may influence, the numbers of the two sexes. The male sex, in his opinion, is determined by the predominance of what he calls "la force motrice," which, in short, is a general term, to comprehend all the causes which tend to fortify and increase the active powers of the body, such as healthful exercise, temperance, good nourishment, &c. The co-existence of these circumstances, he considers to be decidedly favourable to the procreation of boys; and the coexistence of the opposite circumstances, or of those which enfeeble the body, to be favourable to the procreation of girls. The relations of age, temperament, &c. of the two parents may also have a very considerable influence in affecting the predominance of the male or female sex of their offspring. He has ascertained that throughout the departments of France, where rural industry most prevails, the relative number of male births is above the average; and, on the contrary, that where the inhabitants are either idle, or engaged in occupations which require intelligence rather than strength, and attention rather than activity, the relative number of female births is always high.

With the permission of the prefects, M. Girou obtained authentic registers of the births, during the last ten or twelve years, in the cities of Lyons, Marseilles, Bourdeaux, Rouen, Nantes, and Montpellier; and he finds that in every one of these, as in Paris, the number of female births exceeds the average which the entire population of France yields. The statistic reports, taken from many preceding publications, afford similar data. At the Cape of Good Hope, we are told that the masters procreate more girls, relatively, than the slaves; and the registers of hospitals shew, that there is a greater relative number of boys born "hors mariage" than in wedlock. This last observation is founded on numerous statistic reports on the population of Paris, and of the other principal cities in the kingdom, during a period of 14 years.

### Development of the Embryo in Mammiferous Animals.

Dr. Coste, whose researches on the embryology of birds, prosecuted in conjunction with the late Professor Delpech, were crowned with the Academy's highest reward, presented now an account of his Enquiries into the Embryology of Mammiferous Animals. Hitherto, it has been a matter of much obscurity to determine, what are to be considered as the proper ova in them—some physiologists regarding as such the vesicles of De Graaf, while others assert that the ova are the small spherical bodies contained within the vesicles. To determine this very interesting question, Dr. C. examined upwards of forty impregnated rabbits; and the result of these dissections is, that he is inclined to adopt the latter of the two doctrines, and to regard the mammal ovum as, in every respect, analogous and similar to the ovum of the bird.

*Graafian Vesicles.*—These entire vesicles cannot be viewed as ova, for they are much larger in size than the ova which are met with in the Fallopian tubes; for example, in the rabbit, the former are a line and a half in diameter, whereas the latter are not more than the sixth part of a line across.

It is an important fact, that if the ovaries be examined two or three days after conception, it will be found that the number of vesicles which have escaped always corresponds with that of the ova, which have been received into the tubes; and, also, that at those places whence they had escaped, their outer membrane, lacerated at one point only, remains behind, and contributes to the formation of the corpora lutea. This fact, which cannot now be called in question, demonstrates, in a most convincing manner, that the Graafian vesicles are not the proper ova, and, therefore, that we shall err, if we attempt to draw any analogy between these vesicles and the *ova of birds.*

*Ova in Mammiferous Animals.*—There exists on the internal surface of the proper envelope of a Graafian vesicle a membranous-like formation, which lines it throughout, with the exception of one point, where a small spherical body, about a sixth of a line in diameter, will be found. This body is the proper ovum—it is quite transparent, and appears to be composed of the following parts:

1. An outer envelope, which Dr. C. calls the "vitelline," because, like the membrane which incloses the vitellus in the bird, it is in immediate contact with the cicatricula, blastoderma,* or the analogous structure; and also because, being unconnected with the formation of the sanguiferous vessels, it will surround the fœtus and its appendages, without having any union of continuity with them.

2. The vitelline membrane incloses within its cavity a spherical mass, of a yellowish-grey colour, and composed of globules and granules. This mass is evidently the proper vitellus, or yolk of the mammal ovum, for it is upon it that the part analogous to the cicatricula, or blastoderma, rests, and it is at its expence that this part is formed.

3. On the surface of the vitellus, we observe a membranous layer, of a yellowish-grey colour, connected by its internal surface with the external surface of the vitellus, and inclosing it all around.

This arrangement might seem, at first sight, to exclude all analogy with the cicatricula of the ovum in birds, since it, during the early period of incubation, is observed only on the surface of the vitellus, and appears as a small spot or island, formed by a circular lamella. But when we consider that the cicatricula, in a short time after conception, is gradually converted into a complete vesicle, which envelops the vitellus itself, the analogy between it and the vesicle above-described, in the mammal ovum, is at once made obvious. This analogy will be strengthened, if we remember that the omphalo-mesenteric vessels, which are developed in the vesicle, portray most correctly the arrangement of the lateral or blastodermatous vessels in the bird, and that the earliest traces of the mammal embryo appear at a point of the vesicle consisting of globules, which are regularly disposed on each side of a definite axis, in consequence of certain internal movements, similar to those which produce the same phenomenon in birds.

The mammal ovum, therefore, while still in the ovary, like the ovum in the bird, consists of three parts—the vitelline membrane, the vitellus itself, and the vesicle of the germ, blastoderma or cicatricula (all these terms have the same import.) Two days after conception, the ova have penetrated into the oviduct; and, when seen there, they have so strong a resemblance to the small spherical bodies which are found within the Graafian vesicles, that we are forced to believe that these bodies are really and truly the proper ova. In other two days, the ova have reached the cornua of the uterus; as yet, they have no determinate position, but, like a drop of water, or small bubble of air, they are quite free and moveable. At this period, they are about one line in

---

* This word is derived from *βλαστανω*, germinare, and *δερμα*, cutis, and has been applied by Pander to the membraniform body which is situated beneath the cicatricula of the ovum, and whose development produces all the parts of the chick.

diameter, and may be perceived with the naked eye. The vitelline membrane and the vesicle of the germ may be easily made out; but the vitellus has diminished, in proportion to the increase in size of the vesicle. Five days after conception the ova have assumed a fixed position, which they retain during the whole term of gestation. They adhere to the internal surface of the uterus, but as yet this adherence is only that of contact; their diameter is now about two lines; their form is spherical. The vitelline membrane has meanwhile increased in volume, much more in proportion than the germinal vesicle which it incloses: this latter occupying only a third of the bag formed by the membrane. It (the vesicle) retains all the characters which it presented, while yet in the ovary: it adheres by one point of its surface with the inner face of the vitelline membrane, at the spot where this last is applied to the uterus. At that point it exhibits a circular, or elliptic spot, produced by the assemblage of the globules, which we have previously alluded to. This spot Dr. Coste considers to be the rudiment of the embryo; it is seated, he says, in the superficies of the tissue of the germinal vesicle.

The two important deductions which Dr. C. draws from all his inquiries are, 1st. That the small spherical bodies contained within the Graafian vesicles are the proper ova in mammiferous animals; and, 2ndly. That these ova are quite similar to those in birds.

---

### XVIII. LACERATED PERINEUM, TREATMENT OF BY OPERATION.

By far the most common cause of division of the perineum in females, is the injury sustained during a difficult labour; and this injury may proceed either from the excessive distention of the part, when the head of the child is making its escape, or from the mal-application of an obstetrical instrument, as of the forceps, lever, &c. Occasionally indeed this accident has arisen from an outward wound, or from a spontaneous and gangrenous ulceration; and in addition to these causes we may also mention, that a most complete destruction of the perineum has sometimes followed injudicious attempts to cure a fistula of the part. The extent of this injury may be very different; the perineum only may be lacerated; and this laceration may be either complete or partial, the anus and its sphincter remaining entire; or a central perforation may have taken place in the perineum, and we know that in some rare cases, the child has actually been forced through this perforation. In another set of cases, we find that the perineum escapes, and the recto-vaginal septum is lacerated, or destroyed; and lastly, both parts may be injured together. It might very naturally be supposed that, when the anterior part only of the perineum, or as it is called, the fourchette, is divided more or less, the accident would be much more easily remediable, than when the sphincter ani is involved; but the very reverse is often found to be true; for indeed, the re-union effected by nature in the first case is always incomplete, and the female is constantly annoyed, more especially if she be young, with a state of parts, in which the vulva is considerably prolonged backwards, and has lost much of its contractility.

At present our attention will be limited to the most severe accident of all; that in which the whole extent of the perineum has been lacerated, either with or without an injury of the recto-vaginal septum.

This accident seems to be irremediable by unassisted nature; the edges indeed of the wound may cicatrize, but the healing is never accomplished throughout its whole depth. It forms a very frightful calamity; the vagina and rectum are laid into one, and the discharges of the latter are often voided by the former passage. It is however by no means unfrequent, that the female becomes again pregnant, and her accouchment may be the more easy and rapid.

M. Roux knows an English lady who suffered a complete laceration of the perineum in her first labour, and afterwards gave birth to twelve children successively, the accident remaining unrelieved all the time. He thinks it very probable that a great many females may be the subjects of this disgusting calamity, who are ashamed to avow its existence.

In the worst cases the state of the patient is truly miserable; the power of retaining the fæces, &c. may be utterly lost, and she is sometimes constantly harassed with the desire of evacuation, and before she has time to prepare for it, the vagina and adjacent parts may be in a moment deluged with it. She is thus forced to seclude herself from all society, and it cannot be surprising that her general health soon languishes and decays.

How gratifying must it be to a feeling surgeon to be able to rescue a fellow being from such distress! The earliest case on record, where an attempt was made by the surgeon to repair the loss of the perineum, is one which occurred to Guillemeau, the disciple of Ambrose Paré; the interrupted suture was employed,, and the operation was quite successful. Subsequently to his time, the operation, although spoken of and recommended by some writers, was seldom or never attempted, until about the close of last century; when two French surgeons, MM. Noel and Saucerotte, performed it with complete success, by means of the twisted suture. Since that period it has been put in practice about half a dozen times in France; but in most of the cases, with little or no benefit to the patients. The English surgeons seem to have altogether neglected making any attempt in this field of surgery; and the Germans, though of late they have been ample in their descriptions of the best method of operating, have not contributed any essential improvements. M. Dieffenbach, of Berlin, has been most zealous in the cause; but with some of his opinions we cannot agree; he tells us that there is no chance of our being able to effect a complete union of a divided perineum, unless we previously make two parallel incisions along the sides of the vulva and perineum, in order that the parts may yield, and thus allow themselves to be kept in easy and natural contact. We shall see hereafter that this preliminary step is quite unnecessary, and ought therefore to be altogether abandoned.

It was the following very interesting case which suggested to M. Roux, that improvement in the operation, from which he anticipates the most agreeable results in future.

*Case* 1. A young lady, 22 years of age, came from Normandy to Paris in December, 1831, for the purpose of having M. Roux's advice respecting a division of the perineum.

She had been married to a medical man, when she was only 19 years old; and very soon after marriage had become pregnant, so that her accouchement came on just as she reached her 20th year. The labour was a painful and protracted one, and required the use of the forceps for its completion; unfortunately the perineum throughout its whole extent was lacerated, and the recto-vaginal septum, for about half an inch, was also torn. This distressing accident was now of two years' standing; and nature had done nothing to repair the injury. M. Roux, on examining the parts, found that the division was exactly in the median line of the perineum; its edges or lips were quite smooth, soft, and free from any callosities; so much so indeed that a person might have supposed at first sight that it was a congenital defect. The anus and vulva formed but one common outlet; and hence the condition of this interesting patient was most loathsome and afflicting. In order that the frequent desire of voiding the intestinal discharges might be lessened, she had long accustomed herself to take different preparations of opium; and the effect of these had been, at least in one respect, most soothing; for by regulating the doses she could retain the bowels in a constipated state, for almost any length of time; but notwithstanding this relief, the patient was so afraid of the desire ever coming on unexpectedly, that she quite secluded herself from all society, and her life was spent in wasting melancholy. Fortunately her constitution was decidedly good, and the circumstance of her having acquired the power of confining the bowels, for almost any period which might be desired, was favourable to the success of any operation. The operation was performed in January, 1832, and as M. Roux had at this time no experience in such cases, he followed the practice which he knew had been recommended by most surgeons. The suture which he employed was the twisted one. After paring very carefully the edges of the cicatrized lips of the fissure, he transfixed them with four long needles, introducing these at least one inch from the edges of the wound, so as to prevent the risk of their being loosened by ulceration. No lateral incisions were made, because the part did not appear to be much stretched.

There was not an unfavourable symptom after the operation; the urine was drawn off by the catheter—the most strict regimen was enforced, and the bowels did not act. On the 7th day, M. Roux determined to remove the needles, as the appearance of the wound indicated a re-union throughout its whole extent; but, most unfortunately, this appearance was fallacious, and the adhesion was nothing but a simple agglutination. Two days after the removal of the needles, the wound was quite disunited, and the part, in the course of a short time, was in the same condition as it had been before the operation.

A second attempt was resolved upon, and the patient, although naturally enough disheartened by the failure of the first, was too anxious to submit to any rational experiment, which promised a chance of relieving her from her miserable state.

M. Roux, from reflecting upon the first operation, was inclined to attribute its failure to the employment of the twisted suture, which, acting almost solely on the outer edges of the wound, did not keep up an accurate contact of the parts more deeply seated. That a union did not take place, therefore, no one need be surprised, especially when it is considered that the parts were continually kept moistened with the vaginal discharges.

The preliminary steps of the second operation were quite the same as those in the first. Four strong double ligatures were passed through the lips of the wound by means of curved needles, introduced on the one side from without inwards, and on the other from within outwards; and two pieces of bougie were then laid along the two edges, and accurately retained in their position, the one being received into the loops of the ligatures, while their loose ends were tied firmly over the other. Those who have employed the quilled suture, know that a wound always gapes somewhat after its application, and for a very obvious reason—because the pressure is exerted chiefly on the deeper part, and very partially on the outer edges. M. Roux, having calculated on this, took the precaution of inserting a single fine silk ligature along with each double one; and, when he had adjusted the quilled suture, he then tied these single ligatures as after ordinary operations. On the seventh day, the pieces of bougie, and, at the same time, all the ligatures were withdrawn, and the agreeable discovery was made, that a firm and solid union had taken place. Every day successively, for 10 or 12 days, the consolidation of the part became stronger and more secure, and the bowels, most fortunately, were not once disturbed until the 22d day after the operation; and, although the evacuation then was copious, and of a hard consistence, and accompanied with so much pain and forcing down, that it was necessary to assist its expulsion by pressure of the finger within the vagina, the reunion of the parts had by this time become so complete, that no injury whatever was sustained.

[It would certainly have been prudent to have obviated such a state of things by emollient enemata.—REV.]

At the period when this patient left Paris, there was still a small aperture of communication between the rectum and vagina, immediately above the sphincter ani; the fæces did not, however, pass through it, and M. Roux was informed afterwards that it quite healed up.

The result of the preceding case has been most gratifying; for it appears that the patient was speedily restored to the enjoyment of connubial intercouse, and within five months after the operation became pregnant, and was, in due time, delivered safely of a full-grown child, the perineum escaping entire and uninjured.

*Case* 2. A girl, 21 years of age, was admitted into the Hospital de la Charité in March, 1833. She had become a mother nearly two years before, and so severe had been the delivery, that the perineum had been torn completely through.

Before undertaking any operation, M. Roux subjected her to a very spare diet for several days, in order that there might be little occasion for relief of the bowels. The steps of the operation were the same as we have described in the former case, and a similar after-treatment was rigidly followed. But, as it could scarcely be expected that the bowels would be quite so accommodating, in the present instance, as not to act until two or three weeks elapsed, and until the union might, therefore, be solidified, M. Roux, on the evening of the 6th day, ordered an emollient enema to empty the gut. On the following morning, the ligatures and bougies were removed, and it was found that a very satisfactory union had taken place. By the end of the third week, the adhesion was perfect throughout, except at the deepest part of the recto-vaginal septum, where a small fistula remained, and gave exit occasionally to intestinal gas; but this also gradually contracted, and had become quite minute, when the girl left the hospital.

The third case occurred in a woman, 29 years of age, mother of five children; her last accouchement had been lingering and severe, and the application of the forceps had induced a complete laceration of the perineum. M. Jacobson, of Copenhagen, and many of the most eminent surgeons in Paris, were present at the operation performed by M. Roux on this woman, and they had afterwards an opportunity of ascertaining the admirable cure that was effected.

The last successful operation was performed on a lady of rank, mother of three children, who had suffered from this distressing calamity for upwards of two years, but who had never mentioned its occurrence to any one, except her accoucheur, until she consulted M. Roux. The steps of the operation were quite the same as he had followed in the other three cases, and a most complete success rewarded his attempt.

The only instance in which he has failed occurred very recently; the patient died on the tenth day. The particulars of the case are worth recording.

The woman was forty years of age, and, in her, the destruction of the perineum had been the result, not of a difficult labour, but of an attempt which had been made by a surgeon to cure a fistula ani, communicating with the vagina. In consequence of this, the parts must necessarily have been more or less diseased, and, moreover, the patient laboured at the time under a complete prolapsus of the rectum: whenever she stood erect, or coughed, sneezed, or in any way exerted herself, the inverted gut was forced through the large gap in the perineum, forming a tumour as big as a man's fist, it might, indeed, be returned, but no means that had been used could keep it up permanently. When this patient entered the Hôpital de la Charité, she was suffering from continued fever, accompanied with diarrhœa, distress of the abdomen, and other symptoms, which indicated some inflammatory disorder of the mucous surface of the bowels. During a period of four weeks, an appropriate treatment quite recovered her; and then, at her own earnest request, M. Roux proceeded to the operation. Unfortunately, on the third day afterwards, fever again set in, the abdomen became very tender, and the diarrhœa returned. The wound did not exhibit any appearances of the adhesive process, and the ligatures had caused ulceration. On the seventh day, the bougies and threads were removed, and, on the ninth, the disunion was complete; on the following day she died. It is quite reasonable to suppose, that the irritation of the ligatures reproduced those symptoms of intestestinal disturbance which ultimately proved fatal; and M. Roux is candid enough to avow, that perhaps he did not delay the operation for a sufficient length of time after the first illness.

In conclusion, M. R. offers some remarks as to the proper period after the occurrence of the accident for the performance of the operation. When it has taken place during parturition (and this is by far the most frequent cause), it would not be judicious to attempt by art the union of the laceration for at least two or three months; the highly nervous and impressionable state of constitution in a parturient woman, the recent extreme distension of the parts, the copious flow of lochia, &c. are potent reasons against an early operation. Let the wound, therefore, be left to Nature's efforts, until these objections no longer exist, and let the medical man be satisfied with the gentlest treatment.

It will be found that, in most cases in which the operation is performed, a very considerable degree of dysuria takes place for some days; the catheter ought, therefore, to be carefully introduced twice, or oftener, a day. In every one of M. Roux's successful examples, the lips of the wound close to the anus, or recto-vaginal septum, were found to be disunited, although the rest had healed at the time when the ligatures and bougies were removed. There always remained for a week or more, at this part, a fissure, not unlike that which we make in operating for fistula ani; this fissure gradually, however, contracted, and the anus, into which a small oiled tent should be introduced, quickly recovered its healthy condition. The part of the wound most slow in healing is the recto-vaginal septum, and the progress, in some cases, will be found very tedious, in consequence of the extreme difficulty in preventing the passage of the fæces, or of the intestinal gases, from the gut into the vagina. But even this, in course of time, and with the assistance of the judicious surgeon, will contract more and more until it finally closes entirely.

The success which M. Roux has obtained, in a set of cases which are by no means unfrequent, and which, hitherto, have too often baffled surgical relief, encourages him to hope, that the operation which he has recommended will become as common, and as fortunate, as that of staphyloraphy, which he was the first to perform in 1819, although, since that time, no fewer than 65 cases have presented themselves to his notice.—*Journ. Hebdomadaire.*

---

### XIX. Professor Bouillaud's Opinions respecting Typhus Fever.

"It results (says he) from the 36 cases of typhoid entero-mesenteritis, which were lately treated in the wards of the La Charité, that the inflammatory affection of the lower part of the small intestines, and especially of the clusters of the glandulæ Peyeri, constitutes really and truly the fundamental and essential element of the disease. In every instance, from the very commencement of the morbid phenomena, the local symptoms have clearly indicated the existence of such a phlegmasia; and the typhoid state has been developed under the influence of the entero-mesenteritis, in the same manner as we see it supervene in certain cases of severe phlegmonous erysipelas, of phlebitis, &c. It would not be more reasonable to consider inflammation of the intestinal follicles and of the mesenteric glands as a simple consecutive effect of the typhoid state, than it would be to regard a phlegmonous erysipelas, in the course of which typhoid symptoms were developed, as the result of these very symptoms. Such a doctrine would be, in truth, a 'contre-sens,' pathogenesis. We do not, indeed, deny that erysipelas may occur, in subjects already labouring under typhus fever; all that we contend for is, that the obverse case, viz. where the erysipelas is formed before the explosion of the typhoid symptoms, is by no means uncommon, and the scope of our reasoning is obvious when we assert, that the entero-mesenteric phlegmasia is of an erysipelatous character. To those who gainsay our doctrines, we confidently challenge them to adduce a single well-recorded and well-authenticated case of acute inflammation of Peyer's, and of the mesenteric glands, which did not exhibit in its march and in its symptoms, local as well as constitutional, a most close analogy, if not a complete identity, with that disease, which has been most unfortunately designated by the appellations of fever, typhoid affection, &c. If it be true, as the Father of Medicine has predicated, that 'naturam morborum ostendit curatio,' another very potent argument may be adduced in favour of our pathological tenets, from the success of the remedial measures we adopt. These are eminently antiphlogistic. It may be said that some of the means, as the quinine and the chlorurets (which were used in a few of the cases in the hospital), are very far from being so; but who does not perceive that, at the period when we had recourse to these, the inflammatory affection had assumed what the ancients denominated a malignant, or rather a putrid character, and, therefore, required for its arrest the intervention of certain measures, superadded to those of a strictly antiphlogistic tendency? In the period of the malady to which we are referring at present, there is indubitably a focus of putrid decomposition, which, re-acting on the whole economy, induces great and important changes in the mass of the blood and other fluids, and to counteract the effects of which, a new and paramount indication arises—an indication which is best fulfilled by the use of the chlorurets, both externally and internally, and of certain tonics, especially quinine.

How agreeable it is to find out that the doctrines which we have taught and inculcated for so many years, are in accordance with the experience of some of the greatest men who have gone before us. The illustrious Sydenham, whose authority is so often misapplied and abused in the present day, has admirably pointed out the relations between the phenomena of malignancy or putridity, and certain sorts or shades of inflammatory action:—'Cujus de malignitate opinionis inventio, humano generi longe ipsa pyrri-pulveris inventione lætalior fuit. Cum enim hæ febres præsertim malignæ dicantur, in quibus intensioris præ cæteris inflammationis gradus conspicitur.' The following case will illustrate my treatment of the typhus gravior.

A man was brought to the hospital in the second week of typhus fever, and of such an aggravated character were all the symptoms, that we quite despaired of saving him. The prostration was extreme—the tongue, lips and teeth covered with a black crust—breath very fetid—respiration exceedingly feeble—pulse minute, and very rapid—abdomen distended—slight diarrhœa—surface of the belly and chest exhibited several reddish patches and papulæ (eruption typhoide.) The state of this patient positively forbad the employment of any depletory measures, and the only judicious indication seemed to be, to obviate the putrid symptoms by the use of the chlorurets in drinks, baths, and enemas. In three or four days there was a sensible amendment: a blister was applied on the calf of each leg, and ten grains of the sulphate of quinine sprinkled every day on the excoriated surfaces. The diet was gradually rendered more nourishing, and consisted of broths, soups, fruits, and weak wine and water. This patient ultimately recovered.

Let the preceding case satisfy my opponents, that the same treatment is not uniformly, and to the same extent, followed, in my treatment of fever, without regard to the character of the symptoms, or to the constitution of the patient. By a judicious combination of antiphlogistic and antiseptic remedies, 33 cases out of 36, admitted into our wards, were saved. Such success could not rationally be expected to result from a therapeutics, which inculcated the use of stimulants, purgatives, and emetics. At the commencement, indeed, of the malady, a purgative or an emetic may be administered with advantage; but, if they be of drastic severity, or are frequently repeated, the enteritic evil must necessarily be much aggravated. What confirms me in this opinion is, that I know that M. Trousseau has lately abandoned the practice of giving frequent doses of Glauber's salts in dothinenteritis [the entero-mesenteritis typhoide] as recommended by his master M. Bretonneau. At present he appears to be satisfied with the 'medicine expectante;' for he gives nothing else but the white oxyde of antimony, a substance very nearly quite inert; and yet he assures us, that his success of late has been very great in the treatment of cases of genuine typhus. No doubt much good may arise from merely abstaining from every thing positively injurious, and from not interfering with nature's own operations; but we think that even the late M. Dance, who was very sceptical as to the advantages of the common practice in fevers, could not have withstood such practical evidence as we have adduced in favor of the plan which was followed in the 36 cases, of which no fewer than 33 recovered."—*Journal Hebdomad.*

### XX. Hearing, through the Apertures made by the Trephine.

While watching the effects of the operation of trephining, in several patients at the Hôtel des Invalids, M. Perier, an assistant surgeon, has discovered, or at least has imagined that he discovered, that they were all conscious of a sensation of a very unusual and constant noise in the part. We have seen the following experiments made at the clinique of the Baron Larrey, and in the presence of the philosopher M. Savart. The ears of a patient, on whom the operation had been performed, having been well stopped, while the rest of the head was left unincumbered and free, it was found that the sense of hearing was not at all affected, but that he could still perceive every sound quite distinctly, and the more so, when the sounds were directed perpendicularly downwards on the surface of the cicatrix. Even at a considerable distance sounds could be satisfactorily enough distinguished to enable the person to hold conversation with another. The beats of a watch held at a short distance from the cicatrix, were also made out.

Now if, while performing any of these experiments, the artificial aperture in the skull was well covered and compressed with the hand, while the ears remained plugged, the perception of sounds was immediately obstructed. —*Journal Hebdom.*

Are the preceding statements authentic?—Ed.

### XXI. Purification of Theatres of Dissection, &c.

A special commission was lately appointed for the purpose of ascertaining the best method of disinfecting anatomical theatres of their stench and unwholesome effluvia. They tried a multitude of expedients, but found that the use of simple charcoal powder is much the most efficacious. Some of this powder was blended with and sprinkled over the putrid contents of the bowels one day, and on the next, it was always found that their offensiveness was in a great measure removed; and if the students rubbed their hands well with the charcoal before they washed them, all unpleasant smell was most certainly got rid of. This practice has been tried extensively at the dissecting amphitheatre of the La Pitié Hospital, and from its simplicity and efficacy is now constantly adopted there.

One great advantage of the charcoal is that it is a harmless substance, and that it does not even cause the steel instruments to rust, which unfortunately is apt to be the case, if the preparations of chlorine are used as a disinfecting agent.—*Revue Medicale.*

### XXII. Adulterations of Fecula and the means of discovering them.

It appears that of late years adulterations of this most important article of food have been becoming more frequent in Paris. To detect these frauds, M. Payen recommends that a portion of the suspected fecula be incinerated in a platina, or earthenware crucible, and the residue be accurately weighed. If the fecula be pure, however ill washed, not more than a $\frac{1}{200}$th part of the whole remains behind; and even much smaller the quantity will be found to be, if the fecula be exceedingly fine, and quite free from the least admixture.

In performing this experiment the very slow combustion of the vegetable carbon may be quickened, by adding a minute quantity of nitric acid.

Another method, which is more commonly used, as it sometimes enables us to ascertain the proportions, as well as the nature of the adultering ingredients, consists in attempting to dissolve the suspected article. The quantity of undissolved residue gives very nearly the proportion of foreign matters; and as these usually are either chalk, plaster of Paris, bone-powder, &c. we cannot have much difficulty in arriving by analysis at an accurate knowledge of their nature.

Besides the two methods now mentioned, there is a third, which is the most simple and expeditious of all; it is the examination of the suspected flour with the microscope. We have only to sprinkle a minute quantity on a small glass disc, causing the light to be directed upwards through the thin layer. If the fecula be perfectly pure, we shall see only a number of granules which are rounded, diaphanous, white, and with edges somewhat shaded; if on the other hand, it has been adulterated with either of the three formerly mentioned substances, we shall discover among these granules many which are opaque, of an irregular and angular shape, and having a brown or other dingy colour. Having ascertained so much, we may then proceed to determine the exact proportion of foreign matter in some other way.—*Journal de Chimie.*

---

### XXIII. Encephaloid Disease of the Kidney.

A woman 56 years of age, was admitted into the medical wards of the Hôtel Dieu, under the care of M. Caillard.

She was much emaciated, had a jaundiced hue of skin, and the expression of her features indicated some deep-seated and severe suffering. The abdomen was swollen to about the size of a six-months' pregnancy. Pressure however did not cause pain, except when made on the left hypochondrium, and then the patient complained of uneasiness in the region of the kidneys.

When the left hypochondrium was carefully examined a large and compressible tumor might be discovered in the situation of the spleen, which it seemed to have pushed up against the diaphragm: it also extended downwards into the left iliac fossa, occupying all its extent. At the upper and anterior part, an indistinct fluctuation could be perceived; and from this symptom the case had been pronounced to be one of an encysted swelling; and this opinion was thought to be confirmed by the circumstance of the fluctuation becoming quite distinct in the iliac regions, so that a tap on one side was immediately felt by the hand applied to the other.

The question came to be, what was the exact situation of this supposed encysted tumor. Most of the physicians who had examined this patient concluded that the disease was seated in the left ovary.

M. Caillard, without designating the specific nature of the morbid change, was of opinion, that the kidney was chiefly involved. But it was not so much for this morbid enlargement, as for a troublesome diarrhœa which had afflicted her for the last three months, that this woman sought medical relief, when she was admitted into the hospital.

The following is the report of her health for some years before. At 48 years of age her catamenia had quite ceased, and from that period up to five months ago, her health had been moderately good; but losing her husband at that time, she was so much distressed in mind, that her extreme agitation seemed to be the cause of bringing on a violent uterine hæmorrhage, which greatly reduced her bodily strength: there was a relapse of this hæmorrhage in the course of another month; and it is from the date of this relapse, that she reckons the commencement of her present illness, whose earliest symptoms were sharp darting pains in the belly, a feeling of unusual weight there, and considerable difficulty of breathing, especially after meals. When the diarrhœa supervened, it speedily exhausted her little remaining strength, and she died soon after her admission into the hospital.

*Dissection.* About a quart of yellowish serum was found within the abdominal cavity; the small intestines had been pushed upwards and to the right side, and the descending colon also was situated higher than usual, evidently tilted up by the subjacent tumor. This diseased mass, invested with a large quantity of fatty matter, occupied the space, which we pointed out, when mentioning the symptoms.

Its shape was that of a kidney although at least eight times as bulky as that organ. It was readily detached from the surrounding parts, except at its inner side, where the renal vessels were affixed, and also an appendicula, which terminated in a pouch of the size of a pigeon's egg. On cutting it (the appendicula) through, no cavity was found within; it had the appearance of a condensed cellular cord: this was therefore no doubt the ureter, and the terminating pouch was the pelvis of the kidney. When the whole of the morbid mass was removed, it was found to weigh nearly two pounds and a half. It had an ovoid form, was eight inches long, five across, and about four in thickness. A deep incision having been made from the convex outer to the concave inner side, exhibited the structure of the tumor. The natural appearance of the viscus was not recognizable at any part: the upper third consisted of a mass of softened encephaloid matter, occupying the place not only of the tubular, but also of the cortical substance.

It was probably to this deposit that the indistinct sense of fluctuation during the life of the patient, was owing. The central portion of the mass exhibited a greasy, yellowish substance, resembling lard which had been exposed for a long time to the air. The lower third was in a state of crude scirrhus, and exhibited several bloody cavities, "foyers apoplectiques," around which the scirrhus had become softened. The right kidney and urinary bladder seemed quite healthy; and the other abdominal and pelvic viscera were nearly natural.

*Remarks.* M. Andral has recorded in his work on Pathological Anatomy two cases of encephaloid degeneration of the kidney; and Professor Bouillaud [Art. Cancer du Nouveau Dictionnaire] relates a case in which he found the left kidney enormously enlarged, extending from the spleen into the iliac fossa; and its entire structure converted into encephaloid matter. In this, and also in Andral's cases, the renal veins and the vena cava, for some extent, were filled with a pappy sanious matter, somewhat resembling in appearance the diseased substance of the kidney. The symptoms during life had been exceedingly obscure. M. Cruveilhier, in his great work on Pathological Anatomy, has described a case very similar to that which forms the subject of this paper. The abdomen was tympanitic; and in the left flank there was a large indolent swelling [which however the patient had never noticed himself,] extending from the lower false ribs into the iliac fossa. Five months before death severe pains were felt round the umbilicus, and hæmaturia came on, which lasted more or less for a month; after which time the urine regained its natural appearance. A diarrhœa supervened, and put an end to life. The site of the diseased kidney was nearly the same as in our case; and the viscus when cut through exhibited the genuine "cancer encephaloide," viz. the brain-like degeneration in some parts, and in others, cysts filled either with a limpid serum or with a blackish fluid.

It is a remarkable feature of cases of extensive renal disease, (at least when only one of the organs is affected,) that in their early stages the urine very generally presents sufficiently obvious indications of some existent disease, whereas, in the ulterior stages, this secretion very often regains its natural appearance and qualities. The cause of this may be found in the disorganization, as it progressively increases in extent, gradually abolishing the function of the affected kidney; and in the other one, which may be sound, or may be merely hypertrophied, performing the duties of both. We have already stated that not only may the ureter be involved in the morbid deposit, but that the renal blood-vessels are generally, in the progress of the disease, plugged up, either partially or entirely. The immediate cause of death in most cases of carcinomatous disease is perhaps chronic enteritis; and it is this state which induces the diarrhœa that so frequently proves fatal. The cancerous mischief, at first local, becomes at different periods constitutional, and not only one organ, but many may exhibit the inoculation of its incurable operations. It is singular to observe how very extensive and frightful the local disorganization has sometimes proceeded before any symptoms of general disturbance are developed. A boy, four years of age, but so large and well-formed, that he looked rather like a child of eight, was brought by his parents to the Hôtel Dieu, for a swelling which they suspected to be a hernia; but which proved on examination to be quite unconnected with any abdominal protrusion, and appeared to be some affection of the testicle, or of its membranes. It was first supposed to be a hydrocele, as the swelling was indolent, and communicated an indistinct sense of fluctuation, although no trace of transparency at any part could be discovered. The child's health seemed invariably good, his cheeks being ruddy, and his appetite always vigorous.

As there was a certain degree of uncertainty in the diagnosis, it was judged proper to puncture the tumor with a trocar to ascertain the nature of its contents. On withdrawing the instrument, nothing but a little blood flowed out; and the surgeon being, in consequence of this, apprehensive that the disease was of a more serious character, resolved to extirpate the testicle. The tumor, when examined after removal, exhibited the genuine appearances of an encephaloid sarcocele.—*Revue Medicale.*

### XXIV. Encysted Tumor situated on the Larynx—Treatment of.

A man, 58 years of age, consulted M. Dupuytren respecting a tumor situated over the thyrohyoid space, and which had existed there since he was a child. When the finger was introduced into the posterior fauces, a protuberance could be felt under the base of the tongue, and the opening of the larynx seemed to be considerably encroached upon by it. The tumor outwardly was of a lengthened form, extending backwards and to the right side, and communicated a distinct sense of fluctuation—the skin over it was sound and moveable. Its size was that of a large hen's egg. For nearly thirty years it had not exceeded the size of a walnut, but within the two or three last it had increased rapidly, and had occasioned very considerable inconvenience and distress; for the voice had become so feeble and imperfect, that the patient could with difficulty make himself understood. The respiration too suffered; and at times there were attacks of suffocating dyspnœa. M. Dupuytren pronounced the tumor to be of an encysted nature, stating, at the same time, that as it was congenital, it might possibly contain hairs. He dissuaded the extirpation of the swelling, and was content to make a puncture into it to evacuate its contents, and then to introduce a small piece of linen as a tent into the wound, in order that the secretion from its walls might find a ready exit, and that these might gradually contract and close together. However simple such an operation may appear to be, it is quite possible, considering the importance of the adjacent parts, that it may occasion some troublesome consequences, if inflammation was attacking the opened cyst. The frightful dangers of laryngitis and tracheitis, of œdema of the glottis, &c. have not unfrequently supervened on operations on the forepart of the neck.

The patient entered the Hôtel Dieu with the view of having the swelling lanced. The fluid first discharged was clear and of a yellowish colour; but afterwards it was of a thicker consistence, and was found to contain a fatty micaceous matter, which Dupuytren considered as adipocire. When the whole contents had flowed out, the breathing of the patient became immediately much more free, and his voice regained its natutal force and tone. No inflammation came on, and the subsequent suppuration was very trifling. The wound was healed in the course of a fortnight. The patient left the hospital quite relieved. The fluid may indeed re-accumulate, but the same operation will probably be again sufficient. —*Journal Hebdomadaire.*

---

### XXV. Flat-footedness—Evils and Treatment of.

A case will best illustrate the inconveniences which may arise from the sole of the foot being unnaturally and uniformly flattened. A young man, 18 years of age, complained that he could not walk the shortest distance without suffering much pain in his feet, and being almost immediately quite fatigued. If the ground was at all uneven, the distress was the greater, indeed so much so, that he could not walk along the streets of Paris for even one quarter of an hour, without stopping to rest, and to relieve the uneasiness of his soles. On examining his feet, there was no appearance of excoriation, or of any great tenderness of the skin, but it was found that the left sole was flat from the heel forwards, so that the whole surface rested equally and at the same time on the ground; in short, the patient had "un pied plat." He was ordered a shoe made with a higher heel than usual, so that the pressure of the foot might rest on the heel and arch of the toes.

*Remarks.* The term "pied plat" has often been applied as a contemptuous epithet by the higher orders of society to the labouring classes, whose necessities oblige them so often to carry heavy weights, and to submit to other painful and annoying labours.

Now it is very generally true, that by far the greater number, or indeed almost all the cases of flat-footedness, arise from the causes now mentioned; while the person is yet young, and the bones have not acquired their perfect and unyielding hardness, whatever forces him to press the feet with more than ordinary firmness against the ground, as in lifting and in carrying heavy weights, and indeed in any fatiguing employments which oblige him to stand, may cause the deformity of which we are now speaking.

Fortunately the treatment of it is at once most simple, and very generally quite effectual. The shoes must be provided with heels, which are from one to two inches high, according to the extent of the evil; the soles ought to be of flexible materials, and not of such an unyielding substance as wood. This easy remedy will not only afford relief to the present inconvenience in walking, but if employed at an early period of life, will cause the foot gradually to assume the proper vaulted form, which will remain unchanged when the bones and ligaments have acquired all their firmness and solidity.

Such therefore is the ready expedient to remove the inconveniences of flat-footedness; and it is somewhat amusing to take notice that this infirmity, which is almost peculiar to the working classes, is cured by the very means which those who used to taunt them, wished to retain exclusively among themselves; for formerly it was considered as a mark of high life to wear very high heels to their "chaussure;" and since these were generally made of a red colour, they acquired the name of "talons rouges."—*Journ. Hebdom.*

---

### XXVI. Dislocation of the Astragalus — incomplete Reduction, and Practice of M. Dupuytren.

A man, 47 years of age, a German by birth, and of a healthy constitution, fell down a flight of stairs one night, when he was drunk. His account, therefore, of the accident was imperfect. He immediately felt a severe pain in the left ankle, and the foot on this side was found to be deformed, and all motion of it was exceedingly painful.

On the following morning, he was taken to M. Dupuytren's consultation, and the following is the report of the case when he examined it.

Foot turned inwards; immediately below the situation of the malleolus internus (which cannot be felt) there is a deep depression—external malleolus very prominent, and below and in front of it there is another projection, which feels unequal and angular; skin at this part much stretched, severely contused, and slightly excoriated. The foot cannot be moved in any direction—most severe pain when the gentlest attempt is made; swelling not very considerable. The foot appears somewhat shorter than the other, and it is carried a little backwards. No disturbance of the bones of the leg can be detected. The case was immediately pronounced to be one of dislocation of the astragalus forwards and outwards, on the calcareum. There might be also a diastasis of the two bones which form the mortice, that receives the astragalus; but this injury could not be made out.

But in what position was the astragalus placed? Did it retain the relative level of its surfaces, or had it become turned over upon itself? Dupuytren was not able to satisfy himself completely on this point.

*Prognosis and Indications of Treatment.*

This dislocation is always a very serious one, from the extreme difficulty, and even the occasional impossibility, of reducing it. In some cases, indeed, the reduction is effected with surprising facility, the extension being made with the hands alone. But such good luck is very rare, and often the best-directed efforts have failed in replacing the bone. When this is the case, one of two things must happen; either the patient remains a cripple, with his foot twisted inwards, and he is scarcely able to move upon it, or a violent inflammation of the parts may ensue, and oblige the surgeon to have recourse to extirpation of the dislocated bone. This Dupuytren has done in two cases, and in both with success—" the patients being perfectly cured, with a shortening, indeed, of the limb, and a slight limping in their gait; inconveniences which all admit to be preferable to those terrible accidents, which have followed the inflammatory constriction of the foot and leg when the luxation has been unreduced, and which have induced some surgeons to recommend amputation of the limb." Even the extirpation of the astragalus has, in some instances, been followed by fatal consequences.

M. Velpeau mentions a case which occurred to him. A merchant, while leaping out of his carriage, fell upon his left foot, and dislocated the astragalus forwards and outwards. There was also a fracture and wound of the integuments at the same time. The astragalus was removed without difficulty; but an emphysematous tumefaction of the whole limb came on, accompanied with alarming nervous symptoms, and the patient died on the fourth day after the accident.

In the present case, the reduction was attempted in the following manner:—

The patient being in the horizontal posture, a folded towel was passed round the left thigh, which was kept bended at a right angle upon the pelvis, and the ends were taken through a ring fastened in the wall, at the head of the patient, and entrusted to two assistants, who were desired to keep up the counter extension. To prevent the trunk moving, another napkin was carried under the shoulders, and made fast. A narrower one was then passed round the instep, crossed on the sole, and secured by several turns in different directions, some being passed behind the heel. The two ends of this napkin were intrusted to assistants, who were to make extension as directed by M. Dupuytren, who stood on the left side of the patient. The extension he ordered to be made at first from without inwards—then from within outwards, at the time when he endeavoured to replace the bones. Repeated attempts were made, but all in vain; the patient was, therefore, sent to bed, ordered to be bled to twenty-four ounces, to have a warm bath, hot poultices to the foot, and two grains of opium to be taken as an anodyne at bed-time.

On the following day, the man was again taken to the amphitheatre, and the reduction re-attempted, but without success; the foot, indeed, yielded somewhat, and the depression above the outer ankle was considerably diminished. The employment of the hot poultices was renewed; and in the course of a few days, when the inflammatory swelling had subsided, the apparatus which M. Dupuytren uses in fractures of the fibula was applied on the outside of the limb, in order to force the foot as much as possible into its natural direction; but the patient could not bear the extreme inconvenience which it occasioned.

When he left the hospital, he had got rid of all pain in the joint; but the foot was turned inwards, and the toes were pointed downwards.

*Reflections.* The astragalus is so strongly secured by short and very powerful ligaments to the os calcis, and the motion of one bone upon the other is so trifling, that it has been only within late years that a dislocation, such as occurred to our patient, has been accurately recognized and described.

Sir A. Cooper mentions the extreme rarity of the accident, and the great difficulty which is generally experienced in reducing it. He recommends the employment of pullies to effect the extension, and gives tartrate of antimony, in nauseating doses, for the purpose of weakening the resistance of the muscles.

Mr. Cline was in the habit of recommending the following method of attempting the reduction. The thigh should be bent at a right angle on the trunk. While the assistants kept up a uniform extension of the leg, he grasped the metatarsus and protuberance of the os calcis with his two hands, and, putting his knee against the outer ankle, gradually forced the displaced bone back into its place.

After the reduction, a long splint was placed along the fibular side of the leg. This manœuvre appears to us to be preferable to the method adopted by Dupuytren.

The total number of cases of this dislocation which have occurred in M. D.'s practice amounts to twelve. In some of these, the reduction was effected, as has been mentioned already, with great ease; while, in others, all attempts have utterly failed, and the patients have remained lame when they would not submit to the extirpation of the displaced bone.

It is worthy of notice, that in the three or four cases in which M. Dupuytren has extirpated the astragalus, he has always found the bone fairly turned over upon itself, except in one, when it was merely displaced.

We cannot, after learning this fact, be much surprised that the reduction is so difficult, or even altogether impracticable. But, even when the astragalus has not become reversed, we shall find that the configuration of the articulating surfaces of this bone, and of the os calcis, suggests a very obvious explanation of the danger of their forcible disjunction and separation. The posterior part of the astragalus forms a claw-like process, which, when carried forwards, rests between the two articulating prominences of the upper surface of the os calcis, and, indeed, is so confined there, that it can scarcely move.

Any attempt, therefore, to force it back into its normal situation has often the effect rather of pressing the point of the process against the spongy substance of the os calcis, than of lifting it out of the groove in which it has become lodged.

But the question naturally arises, how comes it, that in some cases of this dislocation, the reduction is accomplished with the greatest ease?—Dupuytren accounts for this by supposing that all the connecting ligaments have been so torn and detached, that a much freer motion than is common between the two bones is permitted.

When, however, the ligaments are only stretched and not ruptured, then they act on the displaced bone, as girths, or ties, confining it in its new situation.

The reduction may be easy when the luxation is not complete, or when the hooked process of the astragalus is not thrown so far forwards as to slip into the furrow above described, but merely rests upon the posterior articulating surface of the os calcis.

As to the extirpation of the displaced bone, in irreducible dislocations, it appears to us to be decidedly preferable to the continuance of the distortion and annoyance inevitable from the accident. In the cases operated upon by Dupuytren, the patients did well, and had afterwards serviceable limbs. After the removal of the bone, the gap thus produced is gradually filled up, and the extremity of the tibia becomes so united with the upper surface of the os calcis, that the limb, although shortened, is quite able to support its share of the weight of the body.—*Journal Hebdomadaire.*

---

### XXVII. Spina Ventosa of the Hand.

A youth, 18 years of age, presented himself at the consultation of the Hôtel Dieu, to have the advice of Dupuytren about two hard tumours, situated on his left hand; one on the second phalanx of the fore-finger, and the other on the corresponding metacarpal bone. The first of these had been gradually coming for nine years, the other appeared two or three years subsequently. No cause could be assigned for them. The patient's general health was good, and he said that he never had had syphilis.

The tumour on the finger was of the size of a hen's egg, exceedingly hard and unyielding—the investing integuments seemed quite healthy, although much on the stretch. The other tumour was smaller, and did not exceed the size of a hazelnut; but it was equally hard. Neither of them caused severe or frequent pains, and the chief inconvenience was from their size, and the consequent deformity which was occasioned. The opinion which M. Dupuytren formed of these tumours was, that they were either exostoses or spinæ ventosæ.

"I consider them (said he) as specimens of one of these two diseases, more probably of the latter. The disease is seated in the cavity of the bone, and this cavity is in all likelihood filled with a cancerous substance, which has distended the osseous parietes. This will soon force its way through the barrier, and attack the surrounding soft parts; and whenever this takes place, the case will from that moment assume a more serious character. As to the treatment, there cannot be a difference of opinion on that score; extirpation is the only safe remedy. But here an important question arises for our consideration. As all cases of this description are necessarily involved in some degree of ambiguity before the operation, ought we to extirpate only one, or both of these tumours at the same time? It is to be remembered, that the amputation of any one of the metacarpal, and also of the metatarsal bones, is a much more serious operation than the removal of any finger or toe. However small be the portion of a metacarpal or metatarsal bone which is removed, the danger of the operation is very considerably increased.

The advice of some authors, to saw away a portion of their projecting ends, in all amputations of the first phalanges, for the purpose of making a deeper flap of integuments, is most pernicious; and, indeed, its very converse ought to be invariably followed; viz. that the metacarpal and metatarsal bones should be meddled with as little as possible, and only when there is a positive necessity for such interference. Some surgeons have had their minds so haunted with the dread of relapses, in all cases of diseased fingers and toes, that they have recommended that amputation should always be performed at a considerable distance from the seat of the malady; and many a hand, or at least a large part of it, has been cut away quite needlessly, and therefore injuriously."

On the contrary, Dupuytren has generally made it a rule in his practice, never to amputate more of the hand, or of the foot, than was absolutely necessary; and certainly never to saw away any portion of a metacarpal or metatarsal bone, unless it was decidedly diseased.

In the present case, he determined to remove only the finger at first, in order that he might have an opportunity of ascertaining the correctness of his diagnosis before he meddled with the second tumour. The operation was quickly performed and the wound healed speedily. On examining the diseased phalanx, it presented the following appearances. The bone was much dilated and formed a thin globular and friable shell; when cut through, its cavity was filled up with a greyish-white matter, having the consistence of lard, and deposited in a number of small cells or hollows, formed by osseous lamellæ or spiculæ, as delicate and thin as needles. There were no traces of a medullary membrane.

This, therefore, was a case of genuine spina ventosa; a disease which, although sufficiently well characterized, has very often been confounded with other affections of bone, which have scarcely any feature in common with it; many an exostosis has been described by respectable authors under the appellation of spina ventosa; and this, again, has not unfrequently been confounded with osteo-sarcoma.

The distinguishing character of spina ventosa is the secretion from, and often the complete conversion of, the medullary membrane of the affected bone into a new diseased substance, which, as it accumulates, causes the bony cavity to expand, until it forms a rounded protuberance, whose walls thus become thin, and almost like those of a shell. The contained substance is usually of a gelatinous or lardaceous consistence; sometimes, however, it has a chalky character, is of a greyish or yellowish colour, and has a fungoid appearance.

The medullary canal of a bone becomes expanded, as far as we know, only in those cases where a secretion or a growth is thrown out from the medullary membrane, or where this membrane itself becomes swollen and diseased; in proportion as the fungus in the one case increases and the swelling in the other becomes greater, the bony walls are pushed outwards, until they form a visible tumour outwardly. We have a good illustration of these remarks, in the history of the fungus in the antrum Highmorianum. True it is, that the maxillary bone never exhibits that appearance of an osseous network which we see in one of the long bones, when affected with spina ventosa; but to account for this difference, we should look to the difference of texture of the bones, and to the presence of the marrow in one, and the absence of it in the other.

Some surgeons have been inclined to consider spina ventosa as a disease rather of the substance of the bone, than of its medullary membrane; and they have, in confirmation of their views, alluded to the very expansion or dilatation of the medullary cavity which invariably is present; this, they believe, would not be the case, from the mere presence of a diseased growth within, for the growth, in their opinion, would rather cause ulceration of the bony parietes, and make its appearance outwardly, as we observe in cases of fungus of the dura mater; but we should recollect, in reference to such cases, that the fungus is not situated in a medullary cavity, and, moreover, that it is subjected to constant movements, from the pulsations communicated to it from the cerebral arteries.

On the whole, we are inclined to suppose that all cases of genuine spina ventosa, originate in a fungoid disease of the medullary membrane of the affected bone.

---

### XXVIII. M. BOUILLAUD ON FOLLICULAR ENTERITIS, GASTRO-ENTERITIS, OR TYPHUS FEVER.

The term "follicular enteritis" is to be preferred to the one formerly in use, "gastro-enteritis," on account of its more accurately indicating the nature of the existing lesion; for in many cases there is no inflammation of the stomach, the disease being limited to the mucous membrane, and especially the follicular apparatus of the small intestines; and besides, we see every day examples of genuine gastro-enteritis, unaccompanied with the symptoms of typhus fefever. M. Bretonneau has lately introduced the term "dothinenteritis," or furuncular enteritis, and, although we cannot, with strict propriety, admit the furuncular character of the disease of the intestinal glands, the term is not a bad one. Follicular enteritis has the same meaning, and is more simple in its enunciation. The bizarre appellation of "ileo-dicliditis," in allusion to the seat of the disease in the ileum and ileo-cæcal valve, does not at all express the nature of the diseased change, but rather its mere habitat. M. Bouillaud is in the habit of employing the terms of adynamic or putrid entero-mesenteritis, and, in order to localize the chief seat of the morbid change, adds occasionally a special, instead of the general prefix, thus, adynamic ileo-mesenteritis, &c.

Eighteen cases occurred in the service of M. B. at the La Charité, during the Summer months, and of these, fifteen took place in men, and the remaining three in women. This striking difference is, no doubt, attributable to the more debauched and irregular lives of the former, and also to the circumstance of far more men coming to Paris, from the country or elsewhere, for subsistence; for, indeed, the greater number of the cases we see here, are in those who have resided but a short time in the city. Out of our eighteen patients eleven had been only four months in Paris—four from six to twelve months—one fifteen, and another twenty months resident there. They were all under 28 years of age. The season of the year, viz. during the months of June, July, and August, is that when the follicular enteritis is usually most frequently seen. It is always a difficult, and often an impossible thing, to trace the exciting cause of fever to any one specific agent or influence; and we shall be generally correct, if we enumerate, not one, but several, such as recent arrival from the country, laborious and excessive work, unwholesome and acrid food, debauchery, exposure to the inclemencies of the weather, depression of spirits, &c.

The premonitory symptoms are, a greater or a less prostration, general uneasiness, anxiety, want of appetite, feeling of coldness over the surface, thirst, headache, and, in many cases, some degree of purging and nausea, or even vomiting. The headache becomes more and more severe, accompanied with noises in the ears, appearance of flashes of light before the eyes, vertigo, stupor, so that questions are answered slowly and with reluctance, and, in many cases, with delirium. The features are void of animation, except when inflamed during the delirious paroxysm; the eyes become considerably sunk, the nose is sharpened, and the lips are black, and coated with a dark crust. The skin is always hot and dry—the gentlest perspiration is ever a most favourable symptom; for, in all the worst cases, there is a constant aridity of the surface, and only towards the latter stage is it bedewed with an offensive sickening moisture. In some cases, there is an eruption on different parts of the body; this may be either papular, exanthematic, or pustular. A very characteristic symptom of this fever, when severe, is the position or decubitus of the patient in bed—he lies in one attitude, seemingly unconscious of all around him, and, if he moves, it is rather like the rolling of a senseless mass than the voluntary act of an animate being. Complete coma, convulsions, twitching of the tendons, &c. are always very unfavourable signs. The tongue is generally smooth, dry, and red at the point and along the edges, in the early stage; a filthy, yellow-coloured, cheesy-looking crust covers it, and, in the more severe cases, so completely parched is it, that it has the appearance of having been broiled. The lips, teeth, and mouth are invested with a filthy brown or black sordes, and the breath is offensively disgusting. The thirst is always great—sometimes excessive. The gastric irritation is by no means observed in most cases; thus, in our eighteen patients, six only experienced vomitings; and except in one case, there was not any tenderness of the epigastric region when pressed upon. Pressure on the abdomen, especially over the cæcum and ascending colon, produced pain in ten cases. In fourteen, there was well-marked meteorism, or inflation of the bowels; and the degree of this symptom was usually proportionate to the severity of the case. The involuntary discharge of the urine, especially if this be muddy, ammoniacal, and fetid, always prognosticates great danger. The almost uniform coexistence of pulmonary disease deserves our serious notice; in fifteen of our cases there was acute bronchitis, indicated by a dry sibilant, or mucous sibilant râle; in two, severe cynanche existed, and in one a double pneumonia, which caused the death of the patient. The biliary apparatus was not much affected in the majority of the cases. Four of the patients died, and the following are the most striking necroscopic appearances found.

Serosity under the membranes of the brain; substance of the brain more highly injected than usual; consistence nearly normal. The stomach, in all cases, presented at one or more points arborizations of minute vascularity; the texture of the mucous lining was generally softened, and thinner than in health. The duodenum was but little affected. On examining the ileum, there were found, sometimes even from its very commencement, patches of distinct eruption, or of an intense vascularity, with intermediate portions of healthy surface; but these phenomena were always more distinct as we approached its lower or cæcal extremity; at first, or highest up, the glandulæ Peyeri and aggregated follicles were merely swollen and enlarged; then here and there they were found to be ulcerated, the little ulcers having thickened edges, and the subjacent cellular tissue being denuded; as we proceeded lower down, the glandulæ Peyeri became more developed, and, around them, the single or isolated follicles were converted into aphthous-like ulcerations, with red, and even bloody borders; towards the extremity of the ileum, the gut was found to be actually riddled with these ulcerations, which were surrounded with the soft, pulpy, and inflamed mucous membrane. In one case, the surface of the ileo-cæcal valve, and also of the cæcum, exhibited these last-described appearances. The mesenteric glands, adjoining to the diseased portions of intestine, were red, hypertrophied, and softened. Most of the other viscera, as the liver, spleen, heart, &c. presented more or less ramollissement of texture, so that they were easily crushed between the fingers.

As to the *treatment*, it was in all cases of an antiphlogistic tendency—moderate, but not repeated venesection; the application of leeches upon the epigastric and cæcal regions (the *average* number required for each patient was between sixty and seventy in all, or about 16 at four different times); in four cases, cupping was employed to subdue the bronchitic affection. In nine of the cases, blisters were used, sometimes to the thighs, at other times to the calves of the legs. In ten cases, the solutions of the chlorides (which were first recommended in 1826, by M. Bouillaud, as valuable remedies against the intestinal disease, and the consecutive alteration of the mass of the blood), were employed, either by the mouth, or in injections, or, lastly, mixed with poultices and applied to the abdomen. The bed-clothes, also, of the patients were freely sprinkled with them.—*Journal Hebdomadaire.*

---

### XXIX. Fungoid Tumour of the Dura Mater—Extirpation.

Nicolas Guerin, 59 years of age, presented himself in the consulting-room of the hospital, for advice respecting a tumour on the crown of his head. It had existed for several months, was gradually increasing in dimensions, and every now and then gave rise to severe pains. It was of a rounded shape, as big a small walnut, not at all sensitive on pressure, and free from any pulsatory movements. As the skin over it was sound, M. Berard was puzzled to determine exactly its nature.

The ioduretted pommade was ordered to be rubbed daily upon the swelling, and the patient was directed to return to the hospital after a week or two. But, as the frictions had no effect, Guerin consulted some doctor in the country; and he, supposing it to be an abscess, plunged a lancet into it; but, instead of pus, a quantity of blood only flowed out. The hæmorrhage was with difficulty stopped, and he applied for admission into the Hôpital St. Louis. The tumour at this time was of the size of a small egg, and, in other respects, presented nearly the above-described appearances, but the pain had become more frequent and severe. In consequence of the complete absence of all cerebral symptoms, M. Jobert pronounced it to be a cancerous tumour, seated in the substance of the scalp, and advised its extirpation. The tumour was first exposed by a free incision through the integuments, and then carefully dissected from its lateral attachments down to the bone; and only then it was discovered, that it fairly penetrated through several openings in the parietal bones into the cavity of the cranium.

M. Jobert was satisfied by removing all the mass which projected outwardly, and by aftewards rasping the affected bones. The remaining portion of it could be distinctly seen through the diseased openings, alternately raised and depressed by the pulsations of the brain. We have previously said, that there was not the slightest pulsation before the operation; and, indeed, the absence of this symptom had much increased the difficulty of arriving at an accurate diagnosis.

No serious accident occurred after this imperfect operation, and the pains were even abated. In the course of a few days, the fungoid growth began to rise out of the cranial apertures, and speedily to grow over them, so that they were no longer to be seen at the bottom of the large suppurating wound. The pulsatory movements were still, however, very distinct, and bubbles of air oozed out round the edges of the tumour. As it increased in size, the pulsations became less obvious; but still there were never, at any time, symptoms of cerebral disturbance.

The operation was performed on the 1st of June; and, on the 19th of July, the patient left the hospital of Saint Louis, and returned to that of St. Antoine, under the care, once more, of M. Berard.

The report of his case, at this time, states that there is a swelling on the crown of the head, as large as a man's fist, and extending forwards on the forehead, laterally into the left temporal fossa, and about an inch to the right of the median line of the vertex. It projects about two inches above the surrounding skin, is about four inches across in one direction at its base, and about three inches and a half in the other. It is partially divided into two side halves by a deep ulcerated groove, from which a copious suppuration flows out, and on its surface may be perceived several fistulous openings; the probe, introduced along one of these, can be conveyed to a considerable depth, even into the interior of the cranium, and, at some points, the bone may be felt exposed and rough. The remainder of the mass is covered with the integuments, which are moderately healthy. The isochronous rising and falling of the mass, with the cerebral pulse, are abundantly distinct.

Considering all these signs, the nature of the case could not possibly be mistaken; it was too evidently one of fungoid disease of the dura mater. (M. Berard was at this time ignorant of the appearances which had been discovered during the former operation.) One circumstance, however, was calculated to cast some degree of uncertainty upon the opinion which had been formed, and that was, the entire absence of all cerebral disturbance, as of hemiplegia, stupor, convulsions, loss of memory, and so forth, even when firm compression was made upon the tumour. Surgeons may, therefore, draw a useful lesson from the history of this case. M. B. resolved to follow the advice given by Boyer, in his Treatise on Surgery, to remove all that portion of the cranium from which any part of the tumour sprung out, in order that its base may be got at, and entirely extirpated, along with that part of the dura mater to which it adhered.

The general health of the patient was very good, and had not suffered from either of the preceding operations with the knife. There were indeed some enlarged lymphatic glands on the sides of the jaw and of the neck; but the mere presence of these was not deemed a sufficient contraindication to the treatment proposed; for every day we see examples of such enlargements disappearing, when the disease which has produced them is removed. Some might have supposed that the large extent of cranial surface requiring to be taken away by the saw, and the consequent free exposure of the brain, was a strong objection to the operation; but M. Berard called to mind numerous cases on record, in which very ample portions of the cranium had been detached without the loss of life.

Every thing therefore being considered, he determined upon the use of the trephine. The diseased mass was first very freely exposed, by ample incisions through all the thickness of the scalp, and the bone was made bare all round; the trephine was then applied, and six separate crowns of bone successively removed, an interval of bone of about one line's thickness or perhaps rather more, being left between each crown, and nearly the same between the diseased openings, and the near side of the circles: these narrow partitions were then nipt away with cutting pliers. The operation having lasted already for three quarters of an hour, it was deemed proper to defer the completion of it until the following day, for fear of exhausting the patient's strength. The six trephinings had exposed only one-third of the circumference of the tumor; and it was therefore probable that other eight or six applications of the instrument might still be necessary. The wound was dressed lightly, and the patient put to bed; he passed the night tolerably well, and did not express having suffered much pain. On the morrow the operation was resumed, and it was found absolutely necessary to apply the trephine ten times, before the whole mass could be exposed; the bones perforated were the frontal, two parietal, the left temporal and the great ala of the left sphenoid; and the number of perforations on each were, five on the frontal, four on the right parietal, five on the left parietal, one on the squamous plate of the temporal, and one on the sphenoid; in all 16 perforations. The opening thus made was of an ovular shape; its longest diameter being antero-posterior, and measuring five inches; the transverse one measured four inches and five lines. The exposed dura mater seemed healthy except at the hinder part, where it presented several tuberculous granulations of a cancerous appearance: and it was found that the inner table of the skull had been worn away there. The next step of the operation was to detach a portion of this membrane. It was laid hold of, with a dissecting forceps, and a cut made into it on each side with the knife; these cuts were then enlarged with blunt curved scissors, in a semicircular direction, and made to stop at the falx before and behind: one of the branches of the middle meningeal artery sprung at this time; but the hæmorrhage was arrested by pressure with a piece of agaric. The whole circumference of the tumor was now detached, but as it still adhered behind in the middle to the falx, it was necessary to cut this and consequently also the longitudinal sinus across; very little hæmorrhage ensued, because the cavities of the sinus, and also of the extremities of the dorsal cerebral veins were found to be almost quite obliterated.

The whole mass was now easily detached, and when this was done, the surface of the convolutions of the brain invested with the pia mater and arachnoid coat was exposed to the view, alternately heaving and falling isochronously with the actions of the heart. But scarcely a few moments had passed when the patient became quite insensible, and he was seized with convulsions of the trunk and limbs.

Pressure was immediately made upon all the exposed surface, (these symptoms arising, it was believed from the loss of support, to so large a portion of the brain) and fortunately with the effect of very speedily removing this alarming state. A circular piece of agaric was laid on the surface, and over this, pledgets of lint, and then several compresses; and the whole was firmly secured with a few turns of a roller passed over the crown of the head and under the chin, by means of which a moderate degree of pressure was maintained.

This second operation lasted for nearly an hour and a half, and during its performance, a considerable quantity of blood had been lost. The pulse, however, although at the time when the patient was convulsed, it had become quite imperceptible, soon afterwards regained its former force, and the rest of the day, and the following night were passed without any accident, or any cerebral disturbance. But towards morning the patient became delirious; his features were much altered, he had lost his speech, he gnashed his teeth, and every now and then there were twitchings of the limbs. A small bleeding from the arm, and the application of iced-water to the head were ordered; the pulse however gradually became weaker, and death supervened at seven o'clock in the evening, 34 hours after the operation.

*Examination of the Tumor.* It sprung from the outer surface of the dura mater, and had forced its way outwardly through a large irregular opening, whose edges slanted inwards at the expense of the inner table of the bones. Its substance was somewhat like that of the brain, and might therefore be denominated to be cancero-encephaloid. The inner surface of the dura mater was sound throughout.

*Sectio Cadaveris.* The substance of the cerebral convolutions was found to be inflamed, red, softened, and adhering very firmly to the investing pia mater. Between this membrane and the arachnoid, over the greater portion of the upper surface of the brain and extending downwards and backwards as far as the cerebellum, there was a pseudo-membranous exudation. The cervical glands, although enlarged, did not exhibit any traces of encephaloid disease. The other viscera were sound.—*Gazette Medicale de Paris.*

